# Annotation of Siberian Larch (*Larix sibirica* Ledeb.) Nuclear Genome—One of the Most Cold-Resistant Tree Species in the Only Deciduous GENUS in *Pinaceae*

**DOI:** 10.3390/plants11152062

**Published:** 2022-08-06

**Authors:** Eugenia I. Bondar, Sergey I. Feranchuk, Ksenia A. Miroshnikova, Vadim V. Sharov, Dmitry A. Kuzmin, Natalya V. Oreshkova, Konstantin V. Krutovsky

**Affiliations:** 1 Laboratory of Forest Genomics, Institute of Fundamental Biology and Biotechnology, Siberian Federal University, 660036 Krasnoyarsk, Russia; 2 Laboratory of Genomic Research and Biotechnology, Federal Research Center “Krasnoyarsk Science Center”, Siberian Branch, Russian Academy of Sciences, 660036 Krasnoyarsk, Russia; 3 Department of High-Performance Computing, Institute of Space and Information Technologies, Siberian Federal University, 660074 Krasnoyarsk, Russia; 4 Laboratory of Forest Genetics and Selection, V. N. Sukachev Institute of Forest, Siberian Branch, Russian Academy of Sciences, 660036 Krasnoyarsk, Russia; 5 Department of Forest Genetics and Forest Tree Breeding, Georg-August University of Göttingen, 37077 Göttingen, Germany; 6 Center for Integrated Breeding Research, Georg-August University of Göttingen, 37075 Göttingen, Germany; 7 Laboratory of Population Genetics, N. I. Vavilov Institute of General Genetics, Russian Academy of Sciences, 119333 Moscow, Russia; 8 Department of Genomics and Bioinformatics, Institute of Fundamental Biology and Biotechnology, Siberian Federal University, 660074 Krasnoyarsk, Russia; 9 Scientific and Methodological Center, G. F. Morozov Voronezh State University of Forestry and Technologies, 394087 Voronezh, Russia

**Keywords:** angiosperms, annotation, conifer, deciduous, genome, gymnosperms, microsatellites, RNA-seq, repeats, seasonal senescence, Siberian larch, transcriptome, transposons

## Abstract

The recent release of the nuclear, chloroplast and mitochondrial genome assemblies of Siberian larch (*Larix sibirica* Ledeb.), one of the most cold-resistant tree species in the only deciduous genus of Pinaceae, with seasonal senescence and a rot-resistant valuable timber widely used in construction, greatly contributed to the development of genomic resources for the larch genus. Here, we present an extensive repeatome analysis and the first annotation of the draft nuclear Siberian larch genome assembly. About 66% of the larch genome consists of highly repetitive elements (REs), with the likely wave of retrotransposons insertions into the larch genome estimated to occur 4–5 MYA. In total, 39,370 gene models were predicted, with 87% of them having homology to the *Arabidopsis*-annotated proteins and 78% having at least one GO term assignment. The current state of the genome annotations allows for the exploration of the gymnosperm and angiosperm species for relative gene abundance in different functional categories. Comparative analysis of functional gene categories across different angiosperm and gymnosperm species finds that the Siberian larch genome has an overabundance of genes associated with programmed cell death (PCD), autophagy, stress hormone biosynthesis and regulatory pathways; genes that may play important roles in seasonal senescence and stress response to extreme cold in larch. Despite being incomplete, the draft assemblies and annotations of the conifer genomes are at a point of development where they now represent a valuable source for further genomic, genetic and population studies.

## 1. Introduction

Gymnosperms originated approximately 360 million years ago (MYA), when they comprised a prevailing part of the terrestrial vegetation on the earth [1,2,3]. Today’s living gymnosperms comprise about 1000 species [1], with conifers being the most diverse and abundant group. Being one of the most ancient groups of seed plants, they are considered as a link between angiosperms and pteridosperms. The conifer genomes have a number of features that distinguish them from other plants, the most notable is their enormous genome size, which is not a result of recent polyploidization. It varies among the sequenced species from 4 Gbp in *Gnetum montanum* [4] to 31 Gbp in sugar pine, *Pinus lambertiana* Dougl. [5], much larger than compared to the typical diploid angiosperms, such as 135 Mbp in dicot *Arabidopsis thaliana* [6] or 3.1 Gbp in dicot sunflower *Helianthus annuus* [7], but comparable with some polyploid angiosperms, such as 14.5 Gbp in allohexaploid common wheat *Triticum aestivum* [8] or even smaller, if compared with 150 Gbp in octoploid monocot *Paris japonica* [9]. The gene number also seems to vary in the sequenced conifers, as the number of predicted gene models ranges from 39,370 in Siberian larch, *Larix sibirica* Ledeb. (this study) and 50,172 in loblolly pine, *Pinus taeda* L. [10,11], to 102,915 in white spruce, *Picea glauca* (Moench) Voss [12].

The difference in the conifer genome size has not been shown to be associated with recent polyploidization or a whole genome duplication event [13], however there is a higher gene copy number in gymnosperms than in most angiosperm species, which may be associated with transposed and dispersed duplication events [14]. Another characteristic of conifer genomes is a large proportion of repetitive DNA, estimated as much as 70–82% [10,15,16,17] of the genome size. It is assumed that insertion and extensive proliferation of transposable elements (TE) were mainly responsible for such enlarged genomes [18]. These factors, the giant genome size and its high complexity due to the prevalence of repeat content, make studies of conifer genomes more challenging than in many other plant species.

Owing to the rapidly developing high-throughput sequencing technologies, eleven conifer species in the Pinaceae family have been sequenced, and their draft genomes have been made available to the community, including those for Norway spruce, *Picea abies* (L.) Karst [19]; white spruce, *P. glauca* [12]; loblolly pine, *Pinus taeda* [10,11]; sugar pine, *P. lambertiana* [5]; Douglas-fir, *Pseudotsuga menziesii* (Mirb.) Franco [16]; European silver fir, *Abies alba* [20]; Siberian larch, *Larix sibirica* Ledeb [21]; Japanese larch, *L. kaempferi* (Lamb.) Carr. [22]; Chinese pine, *P. tabuliformis* Carr. [23]; Engelmann spruce, *P. engelmannii* Parry ex Engelm. (NCBI BioProject PRJNA504036); and Sitka spruce, *P. sitchensis* (Bong.) Carr. (NCBI BioProject PRJNA304257).

The Siberian larch is a cold-resistant deciduous conifer tree native to the east and northeast of European Russia, the Urals, and Western and Eastern Siberia [24]. It forms extensive conifer forests, often growing together with Scots pine, Siberian spruce, and Siberian stone pine, occupying almost 263 million hectares, or about 40%, of Russia’s forested areas. The Siberian larch is known for its frost-hardiness, relatively fast growth, and its rot-resistant timber, which makes it especially valuable in construction. Its ecological and economical importance has stimulated exploration of its population structure [25,26,27] and the development of early genetic markers [28,29]. The whole-genome sequencing made possible the development of additional highly informative species-specific SSR markers in *L. sibirica* [30,31], which can be used in different practical applications, including tracking the timber origin to fight illegal logging [32]. The release of the first nuclear [21], chloroplast [33] and mitochondrial [34] genome assemblies for Siberian larch, and recently for Japanese larch [22], has contributed to the development of the genomic resource for the larch genus. Here, we present an initial annotation for the draft Siberian larch genome assembly.

## 2. Results

### 2.1. Transcriptome Assembly

To provide RNA-seq support for gene prediction, five tissues (buds, needles, cambium, seedling, and the first-year shoot) were sampled from a reference Siberian larch tree and used to construct a reference transcriptome. The total RNA was sequenced, using the Illumina MiSeq platform (Illumina, San Diego, CA, USA). All of the clean reads, trimmed with Trimmomatic (9-bp headcrop, minimum read quality of Q = 23, and minimum read length of 35 bp; [35]), were used for the transcriptome assembly. A total of 46,618; 626,542; 59,317; 174,805; and 590,240 transcripts were obtained for the buds, cambium, needles, seedling, and the first-year shoot tissues, respectively, using the TrinityRnaSeq package [36]. The N50 length and average read length of the assembled sequences were 357–790 bp. The reads for all of the tissues are deposited at NCBI SRA under accession numbers SRX9464971, SRX14986114, SRX14997110, SRX14997111 and SRX14997112, respectively. The transcriptome assemblies are available under accession numbers GIXH00000000, GJYD00000000, GJYL00000000, GJYN00000000, and GJYW00000000.

### 2.2. Repeat Content

The species-specific de novo repeat library, generated using RepeatModeler [37], contained 1721 mobile elements that were found in the current assembly of the Siberian larch. Assembling the consensus sequences of ~21 million clusters (cluster size threshold of 200 reads per cluster) with Inchworm from TrinityRnaSeq package [36] resulted in ~31,000 consensus sequences that likely represent the repeated regions of the Siberian larch genome. To validate these sequences, we compared them to the RepeatModeler-derived library and to a PIER repeat library. Homologs were found for ~12,000 consensus sequences among the RepeatModeler-derived library, and for ~7000 sequences among the PIER database. Reciprocal BLAST showed that 1045 out of the 1721 RepeatModeler-derived sequences had a close homology to the clustering-derived consensus sequences. The separate species-specific RepeatModeler-derived library, as well as the combined custom repeat library used for TE identification, are deposited in figshare with DOI 10.6084/m9.figshare.19785913 or can be also found at https://hpccloud.sfu-kras.ru/owncloud/index.php/s/GMBabOGEgqOD4JX (accessed on 12 July 2022).

The proportion of the classified families observed in the Siberian larch genome was similar to those previously described for other conifers. The total number of repetitive elements (REs) in the genome assembly, identified using the RepeatMasker [38] with combined repeat library, was 20.9 million with the total size of 4.8 Gbp, which comprises about 40% of the 12 Gbp genome (Table S1, Supplementary Materials). The fraction covered by repeats in the portion of the Oxford Nanopore long reads was 65.98%, as estimated by RepeatMasker. The rough estimation of the repeat coverage, without considering overlaps, and the nested structure of some repeats was 83.8% (Table S2, Supplementary Materials).

**Figure 1:**
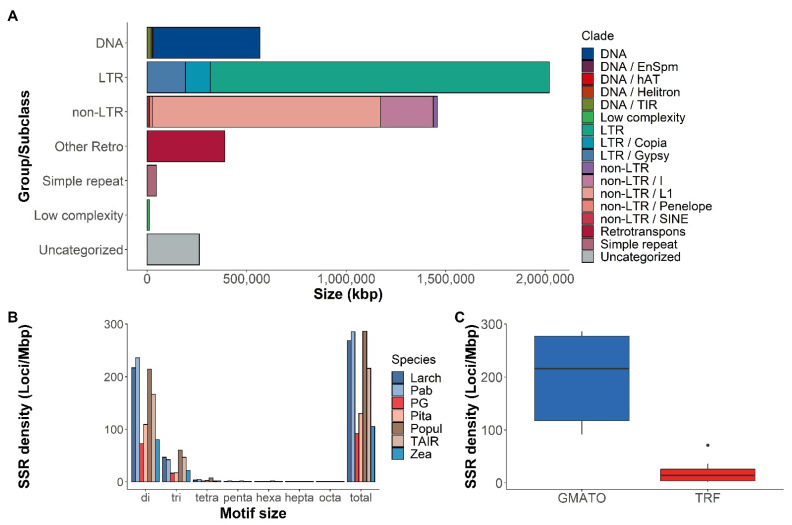
(**A**)—relative size of the repetitive sequence content of the Siberian larch genome annotated using RepeatMasker and combined library, comprising the RepeatModeler-derived library classified with TEclass, RepBase, MIPS, CPRD and PIER v1.0 libraries; (**B**)—microsatellite (SSR) density (number of microsatellite loci with di-, tri-, tetra-, penta-, hexa-, hepta- and octanuclotide motifs per 1 Mbp) for several conifer and angiosperm species found using the GMATo program (Larch—*Larix sibirica*; Pab—*Picea abies*; PG—*Picea glauca*; Pita—*Pinus taeda*; Popul—*Populus trichocarpa*; TAIR—*Arabidopsis thaliana*; Zea—*Zea mays*; (**C**)—box plots for number of all microsatellite loci found in all species listed in B using the GMATo and TRF programs.

The use of the TEclass allowed for a better reconstruction of the TE groups and families composing a large portion of the genome. Among the classified mobile elements, Class I retrotransposons LINE, I, Gypsy, and Copia superfamilies were the most abundant, with LINE elements also having the longest average size and taking the largest part of the genome (Figure 1A; Table S1, Supplementary Materials).

Class I Long terminal retrotransposons (LTR), presented mostly by the Gypsy and Copia elements, comprised the largest fraction of all of the mobile elements. Substantial portions of the LTRs were homologues to a loblolly pine bacterial artificial chromosome (BAC) library and fosmid sequences [39,40]. PtTalladega (3646 copies in the Siberian larch genome), PtOuachita (1025), IFG (990), PtAppalachian (773), PtConagree (731), and eight more repeat families were identified (Table S3, Supplementary Materials). However, most of the LTR-retrotransposons have not been classified into specific families (“Unclassified LTR” in Table S1, Supplementary Materials). Among the non-LTR retrotransposons LINE/L1, I, Penelope, and SINE together comprise about 97.84% of all of the non-LTRs, which cover 11.98% of the Siberian larch assembly length. The majority of the repeats among the different repeat families was relatively small in length, less than 1 Kbp. A small part of the longest repeats reached almost 15 Kbp; they belonged to the LINE elements and uncharacterized LTR. The most frequent TEs for each family were shorter than 1 Kbp. Some of the repeat groups have a bimodal length distribution (Gypsy, DIR, LINE/I, Helitron, Penelope), but both of the peaks in the distributions were less than 1 Kbp (Figures S1–S5, Supplementary Materials).

Class II DNA transposons cover 4.76% of the assembly size, and 4.49% of them were not classified by TEclass (“Unclassified” in Table S1, Supplementary Materials). Among classified transposons the most numerous were terminal, inverted repeats (TIR, 0.16% of DNA transposons), Helitron (0.06%), EnSpm (0.02%), hAT (<0.01%).

In total, 1,129,244 microsatellite loci with motif size 2–8 bp were detected by the GMATo program [41] in the Siberian larch genome, with an average density of 268.7 loci per megabase. Compared to other species, the larch genome assembly also had a relatively high SSR density, similar to the Norway spruce and black cottonwood genomes (Figure 1B). Wegrzyn et al. (2014) and Neale et al. (2014) reported a SSR density of 10–20 loci/Mbp for *Pinus taeda*, *Picea abies,* and *Picea glauca,* discovered by the TRF program [42]. We also scanned the larch genome with TRF, which yielded 17,145 loci with the same motif size and with overall density of 4.1 loci per megabase. On average, GMATo discovered ninefold more SSR loci than TRF, based on seven plant species (Figure 1C; mean 197 and 21 loci/Mbp for GMATo and TRF, respectively), and has proved to be more efficient for the processing of the large genome sequences.

### 2.3. LTR-RT Insertion Time Estimate

**Figure 2:**
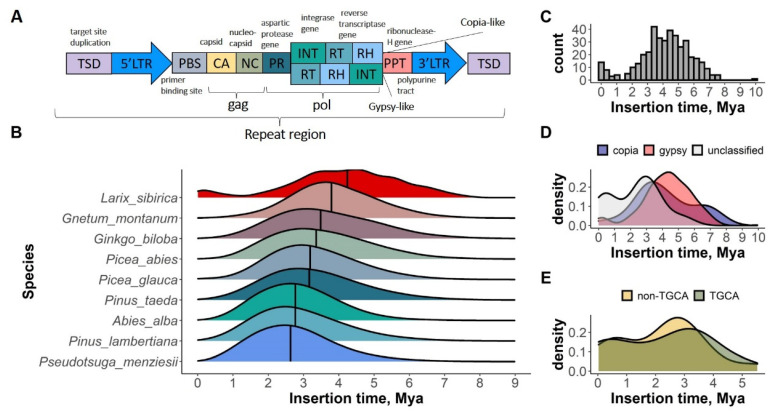
(**A**)—structure of Copia-like and Gypsy-like LTR retrotransposons; (**B**)—estimation of the insertion time of the LTR-RT elements in genomes of nine gymnosperm species; estimation of the insertion time of LTR-RT (**C**); Copia and Gypsy superfamilies (**D**); and TGCA/non-TGCA LTRs (**E**) in the genome of Siberian larch. *X*-axis is in million years (MYA).

The LTRharvest [43] with LTR_retriever [44] identified 347 LTR elements and 36 intact LTRs in the Siberian larch draft assembly. These 36 intact LTRs were combined with 367 identified by Zhou et al. (2021). A possible overlap was checked, using blastn against *Larix* LTRs from Zhou et al. (2021). The probable insertion wave of retrotransposons into the larch genome likely occurred 4–5 MYA, as estimated based on 403 LTRs (Figure 2C). Although the Copia (PR-INT-RT) and Gypsy (PR-RT-INT) superfamilies had slightly different profiles, their mean and median values were very close (mean = 3.16 MYA and median = 3.03 MYA for Copia; mean = 3.11 MYA and median = 2.96 MYA for Gypsy) (Figure 2D). The LTRs with different flanking motifs, typical 5′-TG…CA-3′ and other fewer common variants were also compared in terms of their time insertion. Similarly, the LTRs with TG…CA flanking motifs had a slight peak with a median at 2.56 MYA and LTRs with other flanking motifs had a median at 2.60 MYA (Figure 2E). When compared to the profiles of the other gymnosperms, the larch exhibited the most ancient burst of LTRs insertion, even compared to *Gnetum* and *Gingko* (Figure 2B).

### 2.4. Identification of LRR Genes

In all of the tissues of the Siberian larch, 4482 transcripts containing the LRR domain were detected, using the hidden Markov Model method (HMM) HMMER3 [45] to correctly assign the homologous sequences to one or more Pfam families of LRR. The largest number of LRR domains were contained in the shoot transcriptome (1846), slightly less were in the cambium transcriptome (1599), but their proportions in the total number of transcripts for each tissue (presented in Table S4, Supplementary Materials) were approximately the same for all of the tissues and no more than 2%. The LRR-1, LRR-4, LRR-8, and LRR-6 families encompassed the largest portion of the putative LRR domains identified in the larch transcriptomes (Figure S6A–E, Supplementary Materials). As can be seen in Figure S6A–E (Supplementary Materials), the largest number of transcripts contained the LRR-4 family in all of the tissues of Siberian larch.

The NBS-LRR proteins play an important role in plant defense responses against various classes of pathogens, including bacterial, fungal, viral, nematodes, and insects. Their length usually ranges from ~860 to ~1900 amino acids, but most of the transcripts containing the LRR domain were shorter than 300–400 amino acids (Figure S6F, Supplementary Materials). We filtered out the sequences shorter than 850 and searched for the NBS domain. In total, 56 putative NBS-LRR proteins were found in the transcriptome of the shoot, 18 in the cambium, 5 in the seedling, and 2 in the autumn bud (Table S4, Supplementary Materials). The OmicsBox [46,47] functional annotations confirmed the presence of the domains NB-ARC and LRRs in the identified transcript sequences. The functional annotation by InterProScan [48] did not reveal the presence of other functional domains in these sequences. The NB-ARC- and LRRs-containing sequences in Siberian larch are likely to be resistance genes, because they include P-loop NTPase and LRRs families. The sequences containing identified LRR and NB-ARC domains are deposited in figshare with DOI 10.6084/m9.figshare.19785913 or can be found at repository https://hpccloud.sfu-kras.ru/owncloud/index.php/s/GMBabOGEgqOD4JX (accessed on 12 July 2022).

### 2.5. Structural Annotation Using MAKER2

The benchmarking with the BUSCO package [49] found 317 complete and 307 fragmented genes out of 1614 single-copy orthologs. This makes it possible to estimate the number of complete (not fragmented) genes in this annotation at the level of 32,000 genes, with gene space completeness estimated at 38.6% (Tables S5 and S6, Supplementary Materials). The high fragmentation of the scaffolds could explain the relatively high proportion of the fragmented genes in the Siberian larch genome (19% fragmented vs. 38.6% total) identified in the BUSCO assessment with respect to other conifer genomes (7.5% vs. 80.9% for *Pinus lambertiana*; 11.5% vs. 32.6% for *Picea glauca*).

Using the transcripts from several tissue types, transcriptome shotgun assemblies (TSAs) from other conifer species, and proteins’ references from Uniprot as a starting point for the MAKER2 annotation pipeline [50] allowed us to obtain 39,370 gene models in 37,206 scaffolds, composed of 134,271 exons, and 94,901 introns (Table 1; Table S7, Supplementary Materials). Among them, 24,551 gene models were full-length, and 14,819 were partial (6476 truncated from the beginning, 7545 truncated from the end, and 798 truncated from both sides). The mean length of the genes was about 1841 bp containing 3.41 exons on average, with two being the most common number of exons, which is in a good agreement with prediction of ~four exons per gene for *Pinus taeda* [51]. The maximum CDS length was 7216 bp, which is less than the length of the longest intron of 10,153 bp (Table 1).

The mapping of the transcriptome reads to the genome resulted in 77.9% to 88.8% overall alignment rate per tissue (Table S5, Supplementary Materials). This suggests that the portion of the complete and partial transcriptome-derived genes identified in the genome can be estimated from 21% in needle tissue to 80.4% in shoot tissue.

MAKER2 uses annotation edit distance (AED), a quality control metric initially introduced in the Sequence Ontology project [52,53], where it was originally used to compare and score different releases of the same annotation. Here, instead of assessing the distance between annotations, it measures the congruency between a gene model and its corresponding evidence [50]. For the Siberian larch annotation, the AED computed by MAKER2 was below 0.5 for 95% of the gene models, which is comparable to the mouse genome release GRCm37 and maize chromosome 4 [50]. However, considering the scarce amount of species-specific supporting data that could be used as evidence in the gene prediction and for quality control, this score could be overestimated to some extent.

For the regions identified by RepeatMasker as repeats, intersections with the CDS from the predicted gene models were also found. In total, 6884 genes had at least 20% overlap with a repeat (Figure S7A, Supplementary Materials). Those gene models were consequently marked as ‘repeat associated’; 2247 (33%) of them were overlapping with the Non-LTR I family, 241 (3%) with LINE, 571 (8%) with LTR Gypsy, 523 (8%) with Copia, and 312 (5%) with Simple repeats. The most frequent functional annotations for the repeat-overlapping genes were receptor-like protein kinases, leucine-rich repeat (LRR) proteins, transcription factors, ATP-binding cassette transporters (ABC transporters), synthase, reductase, esterase and peroxidase enzymes, Cytochrome C and Cytochrome P450 proteins, and others (Figure S7B, Supplementary Materials).

**Figure 3:**
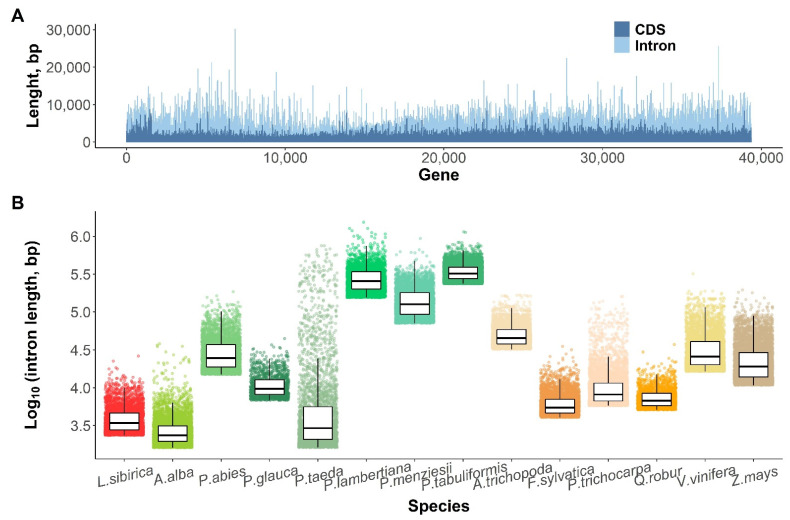
(**A**)—proportion of coding and intronic parts per every gene model in the Siberian larch genome according to the MAKER2 annotation; (**B**)—top 10% of the longest introns across 11 plant species.

Similar to larger genome sizes, the average intron lengths were also longer in conifers than in angiosperms [54]. In the MAKER2-derived annotation, 94,901 introns were identified in the 36,183 genes in total, with an average length of 361 bp and the longest intron of 10,153 bp, which is less than in other conifer species; 289 introns were longer than five Kbp. When comparing the top 10% of the longest introns, the larch introns were comparable in length with those of *A. thaliana* and *P. taeda*, although, the longest larch introns were far shorter than those in other spruce species, such as *P. abies* and *P. glauca*, or in the repeat-rich genomes of *Populus thichocarpa, Vitis vinifera,* and *Zea mays* (Figure 3B). In total, the introns made up to 47% (34,25 Mbp) of the gene space (Figure 3A), and 0.29% of the 12.3 Gbp genome assembly. The repeat content of the introns was lower than in the genome in general; for instance, only 4.59 Mbp (12.9% of the intron sequence space) are covered by TEs compared to 4800 Mbp in the genome (65.98% of genome sequence space excluding introns). In the larch introns, the most abundant were Class I retrotransposons LINE and I (6135 and 2195 elements, respectively), followed by LTR Gypsy and Copia (1879 and 1214 elements, respectively). Among the Class II DNA transposons, the most frequent were the TIRs and EnSpm elements (362 and 245 elements, respectively).

### 2.6. Functional Annotation

**Figure 4:**
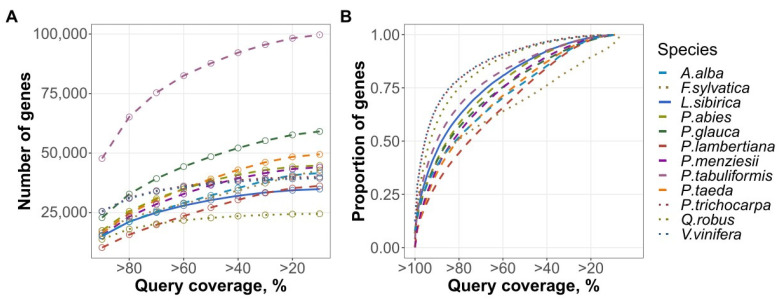
Cumulative number (**A**) and proportion (**B**) of genes aligned to the *Arabidopsis* protein set using *qcovhsp* above a given coverage threshold. Gymnosperm species are presented by dashed lines; angiosperms—by dotted lines; Siberian larch—by the solid blue line.

Based on the sequence similarity to the *Arabidopsis thaliana* protein set, 87% of the predicted larch gene models (34,358 out of 39,370) had an alignment with at least 10^−5^ e-value, 20% query coverage, and 20% identity (Figure 4A). The proportion of the mapped proteins in Siberian larch was among the highest compared to other gymnosperms (second only to *P. tabuliformis*), while being lower than in some model angiosperm plants, such as black cottonwood, grape, and common oak (Figure 4B).

**Figure 5:**
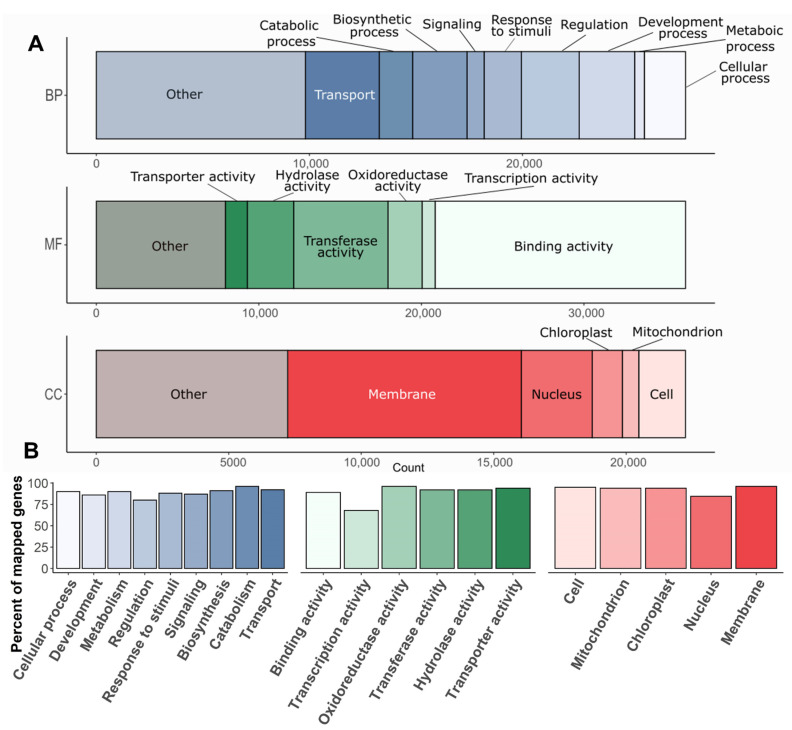
Functional annotation of Siberian larch genes: (**A**)—proportion of predicted larch genes in three functional categories: BP—biological process; MF—molecular function; and CC—cellular component; (**B**)—percentage of larch proteins in different functional categories mapped to the *Arabidopsis* non-redundant protein set with a BLASTP match parameters of e  ≤  10^−5^, pident > 20 and qcovhsp > 20.

The GO category assignment was based on InterProScan domains identification and BLAST homology search, which yielded 30,512 annotated gene models (78%), with at least one assigned GO term. To analyze the annotated gene pool in more detail, it was divided into 20 functional categories. The functions were classified according to the most recent GO dictionary: five categories in Biological process, six in Molecular function, five in Cellular component (Figure 5A). All of the proteins from the respective category were mapped to the *Arabidopsis* protein database with BLASTP and e-value ≤ 10^−5^, pident > 50 and qcovhsp > 50. From 50% (in Transcription activity) to 85% (in Transporter activity, Mitochondrion, and Chloroplast) of the Siberian larch annotated proteins were found to be homologs to *Arabidopsis* proteins (Figure 5B).

### 2.7. Comparing GO Annotations between Conifer and Angiosperm Species

Among the 6937 GO terms shared by 11 species compared in this study, 2080 (29.9%) had significant differences in the number of genes annotated with the corresponding GO term (q-value < 0.005 and adj. *p*-value < 0.01). It was reported recently that the deciduous and evergreen trees differ in the number of genes associated with dormancy and stress response leucine-rich repeats receptor-like kinases (LRR-RLK) [55].

In 12 terms associated with metabolism and the organization of the cell wall and its components, the number of annotated genes was higher in all of the gymnosperms than in the angiosperms (Table S8, Supplementary Materials). For four terms associated with apoptosis and autophagy, the relative gene number in angiosperms and Siberian larch was higher than in the rest of the gymnosperms. Among the 15 terms related to ABA, JA, and ETH metabolism, regulation and response were identified in all 11 species; Siberian larch had the highest number of annotated genes in four of them, compared to both the gymnosperms and angiosperms: jasmonic acid (JA) biosynthetic process (GO:0009695); abscisic acid (ABA)-activated signaling pathway (GO:0009738); ABA binding (GO:0010427); and response to hormone (GO:0009725), respectively. Several of the genes related to the response to JA were generally higher in the gymnosperms than in the angiosperms. In water channel activity (GO:0015250), water transport (GO:0006833), nucleosome (GO:0000786), and nucleosome assembly (GO:0006334), the Siberian larch had a larger number of mapped genes. Among the conifers, the Siberian larch had the highest number of genes in response to light stimulus (GO:0009416) and light harvesting in the photosystem I (GO:0009768) GO categories.

## 3. Discussion

Work with such large genomes as those found in conifers is often hindered by the limit of computational resources, such as the computation time and memory space needed to process the genomic data. The structural annotation of the draft whole-genome assembly of the Siberian larch with MAKER2 pipeline on a 448 core cluster took 22 days to generate a complete set of predicted gene models, and running RepeatMasker separately on the genome assembly of 40 cores took 20 days. This, and the complex genome structure enriched with numerous repeated regions, still makes genomic studies a challenge for plant species with exceptionally large genomes.

### 3.1. Repeat Content and LTR Insertion Time Estimate

There are two main factors responsible for a large genome size in higher plants: polyploidy and amplification of TEs. The latter not only contributes to a genome-size expansion, but also presents a source of genetic variation, increasing the mutation rate and affecting the gene expression by altering coding parts and regulatory regions. A characteristic feature of the conifer genomes is a large number of Res, including TEs. The irreversibility of the repeat accumulation process in the genomes of angiosperms and conifers, also called genome obesity, is discussed in the literature [18,19]. The underlying causes for this are under debate. Some researchers consider this to be a result of bursts in the activity of transposable elements [56,57,58,59,60], while others suggest that the large genomes containing many diverse repeats may have acquired them over time by a steady accumulation process, which may also imply that a repeat elimination process could be slower or less efficient, leading to slow genome contraction [18,61,62].

The types of identified repeats and their distribution in the Siberian larch genome are consistent with those found in other conifers. However, the proportion of the genome represented by simple repeats and mobile elements is one of the smallest among all of the gymnosperms; only 40% of the 12 Gbp genome size can be explained by repeat expansion. This estimate is lower than in all of the other gymnosperms. However, the fraction of the genome covered by repeats in the portion of nanopore long reads was 65.98% bp, as estimated by RepeatMasker, which suggests that the part of the Siberian larch repeatome was too fragmented to be included in the final scaffolded assembly.

LTR comprised the largest fraction of all of the mobile elements with prevailing number of the Gypsy TEs, a characteristic also widely observed in the other conifers [15,16,17,19] and angiosperm species [63]. While the LINEs and SINEs are common for plant genomes, Penelope-like elements (PLEs) were long considered to be a feature of animal and fungi genomes [64,65] until multiple Penelope (EN(+)PLE type or Dryad) elements were found in the loblolly pine [66], and AdLINE3 RTEs were found in a number of flowering plant species [67]. A phylogenetic analysis of Dryad and AdLINE3 suggested a horizontal transfer of TEs, possibly between arthropods and a conifers’ ancestor approximately 340 Mya [66,67].

Class I retrotransposons proliferate by integrating their RNA intermediate into the host genome via retrotranscription to cDNA, using the host transcription machinery and their own enzymes. The coding part of the repeat, placed between two long terminal repeats, contains *gag* and *pol* genes [68,69]. The latter includes protease, reverse transcriptase, ribonuclease-H, and integrase that are responsible for the cleaving of the Pol protein and RNA (protease and ribonuclease H), copying the retrotransposons RNA into cDNA (reverse transcriptase) and integrating the cDNA into the host genome (integrase), respectively (Figure 2A) [70].

In higher plants, the repeats containing direct LTRs prevail [71,72]. When a retroelement has just been inserted, the two flanking LTRs on its 5′ and 3′ ends are identical [73], but, with time, they accumulate mutations, and notably their mutation rate is most likely higher since the repeats, unlike the genes, are not under selection. The number of sequence dissimilarities between the two flanking LTRs can be used as a proxy to estimate the time when the element was inserted into the genome. The estimation of the insertion time of the LTR-RT elements can shed light on the evolutionary aspects of the genome organization, and potentially the date expansion events.

The residual DNA of the LTRs was much higher than the intact LTR-RTs, which suggests that, after a massive proliferation of the retrotransposons, a DNA loss might have occurred in the Siberian larch genome. The typical insertion time estimates in the plant genomes range from 1 to 2.5 MYA for the angiosperms [74,75,76,77,78,79]. In the gymnosperms, 10–15 MYA insertion times have been reported [80]. Based on the LTRs identification by Zhou et al. [81], the approximate time of LTR expansion in the gymnosperms can be estimated to be 2–4 MYA. In the larch, the time estimate can be influenced either by the efficient repeat elimination mechanism combined with the true ancient repeat insertion, or by the fragmented nature of the draft assembly, and consequently, the low number of found LTRs. However, the draft genomes of the Norway spruce and silver fir have comparable assembly contiguity (N50 = 6443, 5206 and 14,051 bp for the Siberian larch, Norway spruce, and silver fir, respectively), and their estimated insertion times are also similar, despite the noticeably different number of identified LTRs (403 in larch, 31,016 in Norway spruce, and 34,952 in silver fir).

Leucine-rich repeats (LRRs) have been found in many functionally diverse proteins. They form horseshoe structures and might be involved in protein–protein interactions. The most representative class of plant-pathogen resistance proteins or R proteins in plants are the NBS-LRR proteins. As their name implies, the NBS-LRR proteins include a nucleotide-binding site (NBS), also called a central nucleotide-binding domain NB-ARC, and a domain containing LRR. The LRR regions demonstrate a high variation in the type and number of the LRR units between and within species, which provides the specificity of pathogen molecules’ recognition. The LRR-containing sequences are associated with immune response of plants to biotic stress [82,83,84,85]. Their further study will also help us better understand the genetic mechanisms of disease resistance in the larch and other plants.

### 3.2. Structural Annotation Using AUGUSTUS and MAKER2 Pipeline

The number of predicted gene models in Siberian larch (39,370) is similar to that in the loblolly pine (50,172) and the Douglas fir (54,830), but much lower than in the silver fir (94,205), sugar pine (71,117), white spruce (102,915), Norway spruce (70,968), and Chinese pine (80,495). Among them, 77% were supported either by RNA-seq data from several tissues, or by homology to the genes from the plant subset of the NCBI *nr* database. The BUSCO analysis suggests that only 39% of the genes had been recovered (Table 1; Table S6, Supplementary Materials). This relatively low percentage can indicate that not all of the genes were fully sequenced and included in the genome assembly. The genome assembly based only on the contigs without gaps covered 5.59 Gbp, about half of the entire genome, while the genome assembly based on scaffolds with gaps covered 12.34 Gbp, which corresponds well to the entire genome. Therefore, we believe that the gaps are not due to the insufficient sequencing coverage, but represent highly repetitive regions that are excluded during assembling procedure, due to their high complexity, while the coding parts of the genome are mostly sequenced and easily assembled, due to their uniqueness. Moreover, comparison to *Arabidopsis* gene set (Figure 5B) shows that a large proportion of the genes have been identified and characterized. Thus, the annotation can still be used as a good resource and a primary reference for further genomic studies.

The average intron length in the larch genome was 1.8 to 3.2 times shorter than in the other conifers. In comparison to the top 10% of longest introns, the larch introns were far shorter than those in other conifer species, such as *P. abies*, *P. glauca,* and *P. taeda* (Figure 3B). The discrepancy could be explained by (1) underestimation of the intron size in the MAKER pipeline [12] that uses the threshold values for resolving the exon–intron structure, or (2) the naturally occurring differences within the conifer clade. The assembly contiguities for *L. sibirica* and *P. abies* are close, as evidenced by their N50 (Table S7, Supplementary Materials; Figure 3B), while their average and maximum intron length differ by 2.8 and 6.7 fold, respectively. Likewise, the average intron size in *L. sibirica* and *P. glauca* were close, differing by 1.8 fold (Table S7, Supplementary Materials), while their N50s differ by 7.2 fold. Nevertheless, it is still possible that the variation in intron sizes in the conifer genome can be explained by a difference in the assembly contiguity.

### 3.3. Functional Annotation

The conifers differ from flowering woody plants in a number of ways, including the absence of vessels in xylem and sieve tubes and companion cells in phloem, different wood structure, haploid megagametophyte (unlike triploid endosperm in angiosperms), megasporophylls, and reproductive structures presented by cones rather than flowers. The conifers, unlike angiosperms, are mostly evergreen plants with very few of them having a seasonal needle abscission (seasonal senescence), particularly in *Glyptostrobus*, *Metasequoia*, *Taxodium*, *Pseudolarix,* and *Larix* genera. The larches are the dominant species in the boreal forests in Western Siberia and Canada, occupying much colder habitats then the other boreal woody plants.

#### 3.3.1. Cell Wall and Phenylalanine Metabolism

The conifers and flowering woody plants differ in their cell wall structure, which influences their woody properties. In woody plants, the common components of a cell wall are hemicellulose, pectin (in the primary cell wall), and lignin (in the secondary cell wall) [86]. In the woody angiosperms, lignin in the secondary cell walls is made up of guaiacyl (G) and syringyl (S) units, while in the gymnosperms, the lignins are homogeneous, consisting primarily of p-hydroxyphenyl (H) and guaiacyl units [87]. It was proposed that the syringyl units have better ability to strengthen cell walls than the guaiacyl units and are advantageous for anti-fungal defenses [88,89]. The primer building blocks for lignin in conifers are p-coumaryl alcohol and coniferyl alcohol [90].

**Figure 6:**
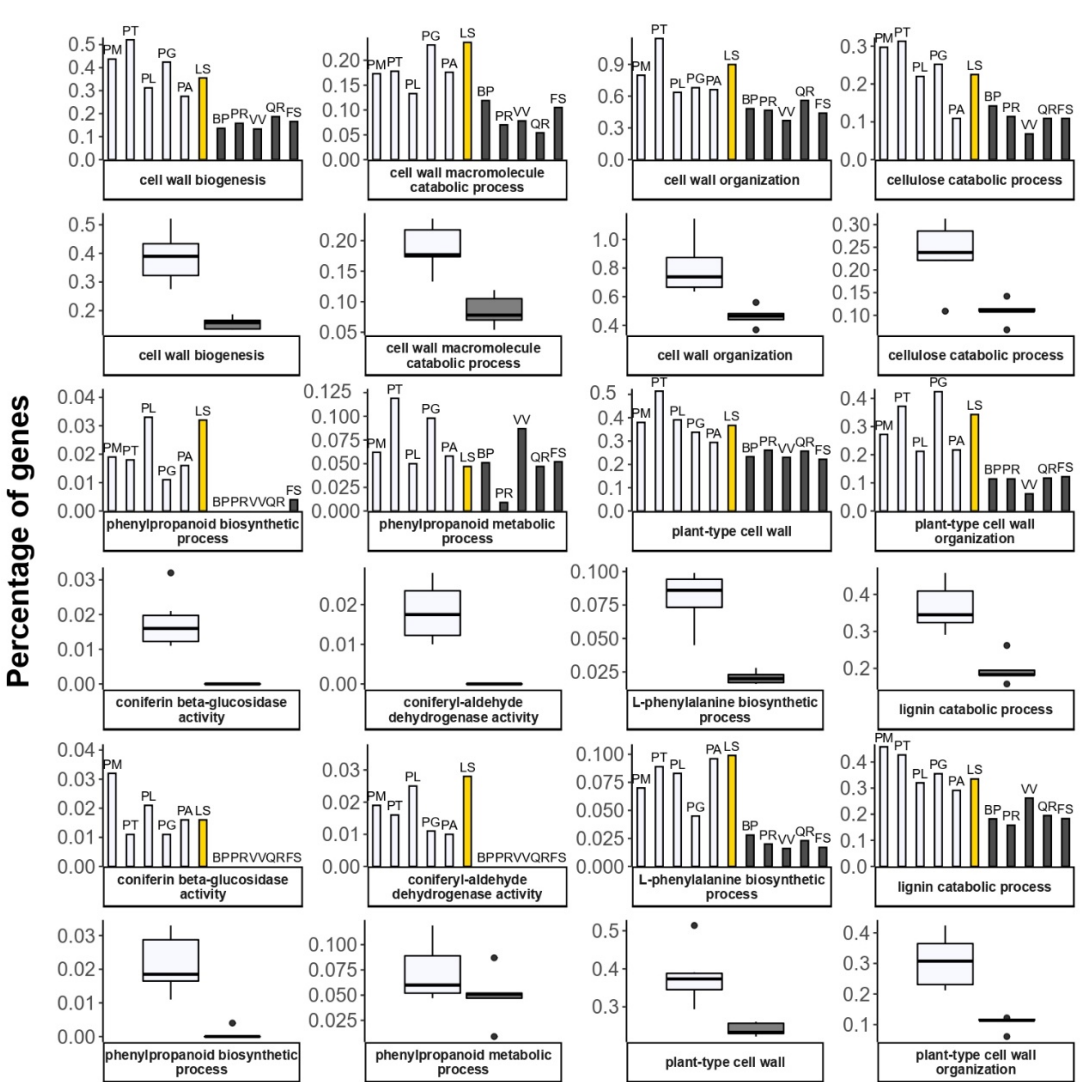
Percentage of genes annotated with GO terms related to cell-wall maintenance. Angiosperm species are represented by solid black columns, gymnosperms by transparent columns, Siberian larch—by yellow. Boxplots demonstrate the difference in gene number between two groups, evergreen (transparent) and deciduous (gray). Angiosperms: BP—*Betula pendula*; FS—*Fagus sylvatica*; PR—*Populus trichocarpa*; QR—*Quercus robur*; VV—*Vitis vinifera*). Gymnosperms: PM—*Pseudotsuga menziesii*; PT—*Pinus taeda*; PL—*Pinus lambertiana*; PG—*Picea glauca*; PA—*Picea* abies; LS—*Larix sibirica*.

The differences between the conifers and angiosperm woody species can be clearly seen in the number of genes in GO terms related to cell wall organization and lignin catabolism (Figure 6). The cell-wall enzymes involved in the biosynthesis of lignin from p-coumaryl alcohol and coniferyl alcohol were identified in all six of the analyzed conifer species.

One of the major precursors for lignin biosynthesis and production of secondary metabolites, such as phenylpropanoids, flavonoids and anthocyanins, is phenylalanine. Conifers utilize large amounts (30–40%) of carbon fixed during the photosynthesis for lignin biosynthesis and wood formation, and their secondary metabolism is remarkably complex [91]. Apart from their building function, phenylpropanoid, in the form of phenolics, terpenoids, and alkaloids, plays an important role in the defense mechanisms against insect and microbial pathogens, functioning as antifeedants and toxins [91,92,93].

The biosynthesis of phenylalanine and phenylpropanoids also demonstrates the difference between the conifers and angiosperms (Figure 6). In plants, the main enzymes participating in the biosynthesis of phenylalanine are prephenate-aminotransferase (PAT), that converts prephenate to arogenate, and arogenate dehydratase (ADT) that transforms arogenate to phenylalanine, that is lastly converted by phenylalanine ammonia-lyase (PAL) into cinnamic acid in cytosol, the first component of the phenylpropanoid pathway [91,94]. It was shown previously that the conifers have more diverse families of ADT and PAT genes, compared to angiosperms [95,96].

#### 3.3.2. Programmed Cell Death and Autophagy

Programmed cell death (PCD, or apoptosis in animals) is an organized and genetically regulated process of cellular suicide that can be induced by external environmental stresses or occurs during the organism’s development. Unlike animals, plant cells have a rigid cell wall that prevents the formation of apoptotic bodies [97], and lack the classical caspases that act as the main inducers of PCD, and phagocytosis or macrophages that could eliminate the remains of a dead cell. Instead, plant cells use caspase-like proteases (metacaspases) to induce PCD [98], and utilize vacuoles and vacuolar-lytic enzymes to digest their cell contents [99,100]. The correct classification of the plant PCD types has been debated [97,101,102,103]. In general, it can be divided into two major types: (1) vacuolar/autolytic/apoptosis-like PCD, characterized by the engulfment of the cytoplasm by lytic vacuoles and the later release of vacuolar hydrolases into the cytosol due to the rupture of the tonoplast; (2) a hypersensitive response PCD that often does not involve swelling of the vacuoles and is characterized by cell shrinkage and increased autophagic activity [102]. The former commonly occurs during the differentiation of the xylem elements, leaf senescence, and megasporogenesis, while the latter is activated in response to pathogen invasion to prevent the further spread of the infection [101]. PCD and autophagy have been shown to be closely related to senescence, since it relies on the degradation and recycling of accumulated nutrients for later use in other parts of organs. Important features of PCD, such as DNA fragmentation and protoplast retraction, were observed during senescence in cucumber [104,105].

**Figure 7:**
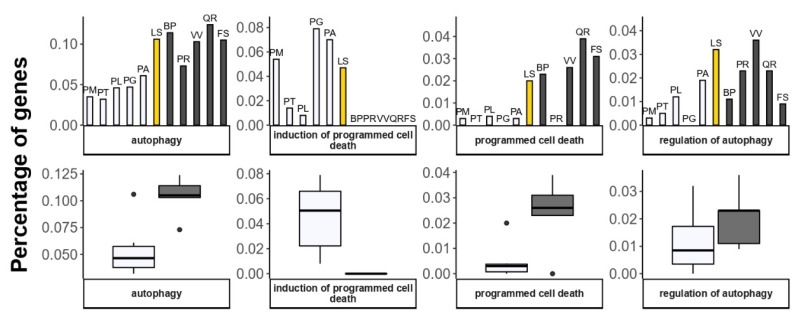
Percentage of genes annotated with GO terms related to programmed cell death (PCD) and autophagy. Deciduous angiosperm species are represented by black solid columns, evergreen gymnosperms—by transparent columns, Siberian larch—by yellow column. Boxplots demonstrate the difference in gene numbers between two groups, evergreen (transparent) and deciduous (gray). Angiosperms: BP—*Betula pendula*; FS—*Fagus sylvatica*; PR—*Populus trichocarpa*; QR—*Quercus robur*; VV—*Vitis vinifera*). Gymnosperms: PM—*Pseudotsuga menziesii*; PT—*Pinus taeda*; PL—*Pinus lambertiana*; PG—*Picea glauca*; PA—*Picea* abies; LS—*Larix sibirica*.

In the GO:0012501 category associated with PCD, the Siberian larch has gene numbers more similar to those in deciduous angiosperm trees, rather than in evergreen conifers. The number of genes associated with autophagy is also higher in the deciduous angiosperms and larch than in other conifers (Figure 7). However, the GO:0012502 (induction of PCD) genes were annotated only for the conifer species (20 in Douglas-fir, 6 in Loblolly pine, 2 in Sugar pine, 42 in White spruce, 22 in Norway spruce, and 12 in Siberian larch).

PCD features, including the altered nuclear morphology and DNA fragmentation, were also observed in tomato leaves and flowers, where treatment with ethylene (ETH) triggered PCD in abscission zone cells, and the application of ROS scavengers delayed abscission [106]. It was proposed that PCD is regulated through the ETH signaling pathway, as ETH-biosynthesis defective mutants exhibit increased leaf longevity [107]. Activation of autophagy during PCD and senescence were demonstrated on autophagy-deficient *Arabidopsis* mutants that demonstrated accelerated senescence and PCD [108,109].

#### 3.3.3. Hormones

ABA, JA, and ETH are phytohormones that play an important role in response to abiotic and biotic stress and leaf senescence. ABA is a phytohormone that participates in a number of processes critical for plant development and growth, such as the control of bud dormancy and seed germination, fruit development, resilience to abiotic stress and pathogens’ infection, and leaf senescence. ABA induces stomata closure, thus reducing water loss via transpiration in response to water deficiency or heat stress. ABA-deficient mutants of *Arabidopsis*, tobacco, tomato, and maize suffer even from relatively moderate dehydration or temperature deviations [110]. ABA is also required for cold-stress tolerance [111], as ABA-deficient mutants of *Arabidopsis* were shown to have reduced freezing tolerance in cold-acclimated plants [112,113,114]. Exposure to low temperatures was shown to increase the levels of endogenous ABA in *Arabidopsis* and wheat. In many plants, ABA is shown to be involved in leaf senescence [115], and together with ETH and reactive oxygen species plays a major role in leaf abscission [116]. In rice and *Arabidopsis* treatment with exogenous ABA was demonstrated to accelerate leaf yellowing and senescence [117,118], and levels of endogenous ABA were reported to be increasing during leaf senescence in maize and *Arabidopsis* [119,120].

JA and its derivatives, referred to as jasmonates (JAs), are fatty acids belonging to the oxylipins family that function as signaling molecules, regulating the expression of genes in response to various abiotic stresses. The biosynthesis of JAs takes place consecutively in plastid, peroxisome, and cytosol, where it is converted from its precursor, α-linolenic acid, through oxo-phytodienoic acid to JA [121]. The process is triggered by abiotic stress that causes an accumulation of JAs in the cytoplasm of stressed leaves [122,123], and activates the JA-signaling pathways. The higher levels of JAs activate the binding of various transcription factors (TFs) to specific jasmonate-responsive genes that otherwise are silenced by the transcriptional repression complex. This complex includes jasmonate ZIM-domain (JAZ) proteins, transcriptional corepressor TOPLESS (TPL), and the novel interactor of JAZ protein (NINJA) [122]. JAs alleviate the effects of water deficiency and soil salinity [124,125,126], low temperature [127,128], excessive UV exposure [129,130,131], and participate in the pathogen-defense mechanisms in gymnosperms [132,133].

**Figure 8:**
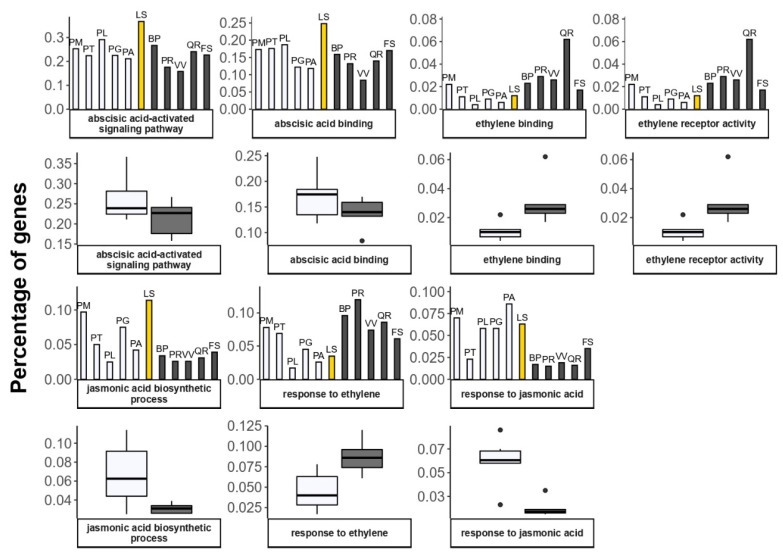
Percentage of genes annotated with GO terms related to hormone signaling and response. Deciduous angiosperm species are represented by black solid columns, evergreen gymnosperms—by transparent columns, Siberian larch—by yellow column. Boxplots demonstrate the difference in gene number between two groups, evergreen (transparent) and deciduous (gray). Angiosperms: BP—*Betula pendula*; FS—*Fagus sylvatica*; PR—*Populus trichocarpa*; QR—*Quercus robur*; VV—*Vitis vinifera*). Gymnosperms: PM—*Pseudotsuga menziesii*; PT—*Pinus taeda*; PL—*Pinus lambertiana*; PG—*Picea glauca*; PA—*Picea* abies; LS—*Larix sibirica*.

The Siberian larch had the highest number of annotated genes related to response to hormones, JA biosynthetic process, ABA-activated signaling pathway, and ABA binding (Figure 8). On the contrary, a number of genes in ETH binding, ETH receptor activity, and response to ETH were relatively lower in all of the conifers, including Siberian larch, than in most of the angiosperm trees. This is probably not surprising, considering that the last components of the canonical ETH signaling pathway emerged after the separation of the angiosperms from the gymnosperms [134,135].

## 4. Materials and Methods

### 4.1. Genome Data

We used a Siberian larch genome assembly v1.0 (NCBI GenBank accession number GCA_004151065.1) with a total length of 12.34 Gb (Table 2), described in detail in Kuzmin et al. (2019) for annotation.

### 4.2. Transcriptome Sequencing and Assembly

The RNA from the Siberian larch buds, needles, cambium, seedlings, and first-year shoots was isolated, using the Qiagen RNeasy Plant Mini Kit (Qiagen, Hilden, Germany) according to the manufacturer’s protocols. The Illumina paired-end (PE) libraries, consisting of 250–400 bp long DNA fragments, were prepared for the larch buds using the TruSeq RNA Sample Preparation v2, and for the needles, cambium, shoots, and seedlings using the TruSeq Stranded RNA Kit with Ribo-Zero Plant kits (Illumina Inc., San Diego, CA, USA). The sequencing was completed at the Laboratory of Forest Genomics, Institute of Fundamental Biology and Biotechnology, Siberian Federal University, Krasnoyarsk, Russia on an Illumina MiSeq platform with 250 cycles in both directions (250 × 2) using the MiSeq v2 Reagent Kit 500 Cycles PE (Illumina Inc., San Diego, CA, USA).

The FastQC software v. 0.11.9 was used to evaluate the quality of the sequencing data (https://www.bioinformatics.babraham.ac.uk/projects/fastqc, accessed on 16 January 2020). The raw sequencing data were processed using the Trimmomatic program v. 0.39 (9-bp headcrop, minimum read quality of Q = 23, and minimum read length of 35 bp; [35]). The SortMeRNA version 4.0.0 was used for the ribosomal RNA removal. In addition, Rcorrector was used for the sequencing error correction [136]. The transcripts were assembled using the TrinityRnaSeq package v2.2.0 [36]. For additional filtering, the transcripts were scanned for the presence of open reading frames (ORFs), using TransDecoder v3.0.1 [137], and conservative protein domains using Pfam [137,138,139]. The transcripts that did not possess coding regions were excluded from the subsequent analysis. For annotation via MAKER2, the pipeline transcriptomes containing rRNA were used.

### 4.3. Repetitive Elements (REs) Analysis and Masking

To search for REs, RepeatModeler v.1.0.11 [37], based on de novo RE detection programs RepeatScout and RECON [140,141], was used. Since RepeatScout does not use all of the scaffolds or contigs for the analysis, but only a random sample, it was decided to only analyze scaffolds longer than 100 Kbp (2869 scaffolds with summary length of 360 Mbp). RepeatMasker open-4.0.6 [38] was used to mask the low complexity regions and REs. The RepeatMasker edition of RepBase 2017.01.27 [142] was extended by larch-specific repeat library, constructed using RepeatModeler open-1.0.8 which was run with default settings. This combined database was used in MAKER2 pipeline to mask the repeats.

To assess the relative abundance of previously characterized repeat families, RepeatMasker was used on the whole genome assembly (12.34 Gbp). The RepeatModeler-derived de novo library was augmented by the clustering of frequently occurring reads from whole-genome sequencing data. The clusters of reads were assembled with Inchworm from TrinityRnaSeq v2.2.0, which resulted in consensus sequences that should represent the highly repeated regions of the larch genome. The unrecognized elements from the de novo repeat library generated by RepeatModeler and the clusters of frequently occurring reads were classified by TEclass v2.1.3, that classifies transposons using the Support Vector Machines (SVM) and LVQ neural network [143]. The combined library, comprising the RepeatModeler derived library classified with TEclass, RepBase library (edition 2017.01.27), MIPS Repeat Element Database library [144], CPRD Custom Plant Repeat Database [39], and Pine Interspersed Repeats Resource library PIER v1.0 [10,39] was used for sequence similarity search. The portion of long reads from the Oxford Nanopore sequencing available for Siberian larch was used to estimate the repeat abundance. In total, 3,060,509 reads with total length = 7.24 Gbp, minimum length = 25 bp, maximum = 77,840 bp, mean length = 2365, min quality = 7, were used to search for known repeat families using RepeatMasker and combined repeat database comprising classified RepeatModeler-derived library, RepBase 2017 edition, MIPS, CPRD, and PIER. The classification of TEs was adopted from the RepBase update [145]. The GMATo [41] and TRF [42] programs were used to search for tandem repeats.

### 4.4. Identification of LRR Genes

The LRRs were searched in ORFs of the Siberian larch transcripts (NCBI SRA accession numbers SRX9464971, SRX14986114, SRX14997110, SRX14997111, and SRX14997112). The ORFs were identified using Transdecoder v.5.5.0 (https://github.com/TransDecoder, accessed on 3 August 2020). The ORFs of the transcript sequences were scanned by HMMER 3.2.1 [45] against the Pfam models LRR-1 (ID PF00560), LRR-2 (ID PF07723), LRR-3 (ID PF07725), LRR-4 (ID PF12799), LRR-5 (ID PF13306), LRR-6 (ID PF13516), LRR-8 (ID PF13855), and LRR-9 (ID PF14580). All of the LRR models were obtained from the Pfam 32.0 database and belong to the LRR clan (ID CL0022). The LRR clan also contains other families, but they were excluded because they represent bacteria, animals, and myxomycetes [139]. A search for NBS R-genes (NB-ARC; obtained from the Pfam 32.0 database: PF00931) was additionally performed, to check if some of the sequences with LRRs belong to R-genes.

The OmicsBox Base Platform [46,47] was used for the BLAST search, GO mapping, annotation, and statistical analysis. Gene ontology (GO) terms associated with the obtained BLAST results were extracted, and evaluated GO annotation was obtained. Enzyme codes were inferred by mapping with equivalent GOs, while the InterPro motifs were directly queried at the InterProScan web service on EMBL-EBI.

### 4.5. AUGUSTUS Training

We used MAKER2 [50] for an automated gene annotation of the Siberian larch genome assembly. The MAKER2 pipeline supports several ab initio gene predictors, including SNAP, GeneMark, and AUGUSTUS [146]. All of them require prior training to obtain species-specific parameters describing patterns of exon–intron structure. We used AUGUSTUS v3.2.1 to generate the preliminary gene models for the large sized genome of Siberian larch. This gene finder is based on a Generalized Hidden Markov Model (GHMM) and demonstrates good performance in ab initio prediction for non-model organisms [147,148], provided it has been trained appropriately, which can be tricky.

**Figure 9:**
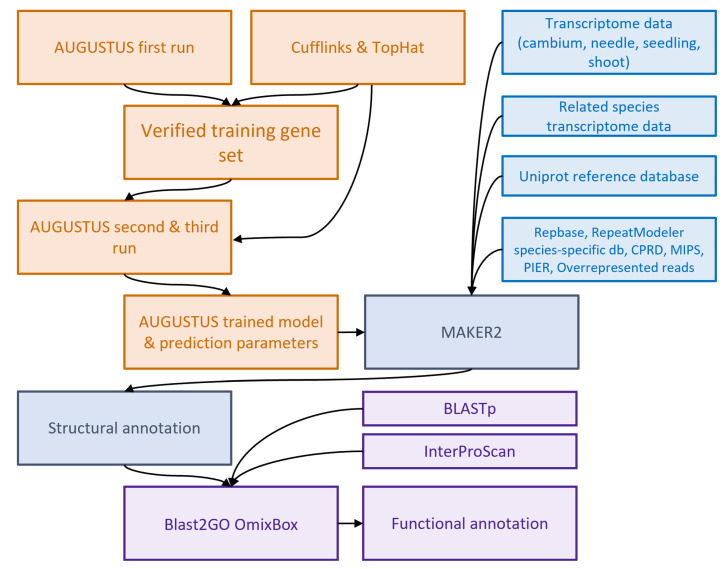
Genome annotation workflow.

The AUGUSTUS training was carried out iteratively in several steps. First, AUGUSTUS was run with pre-calculated parameters for *Arabidopsis thaliana*, which resulted in 916K preliminary gene models and relatively low prediction accuracy. In order to build an initial training gene set, we used RNA-seq data. Transcriptome reads were mapped to the genome using TopHat [149], and the resulting alignments were used with Cufflinks to annotate coding regions [150]. This resulted in 16K transcriptome-derived gene models. Then, two gene sets from AUGUSTUS and Cufflinks, respectively, were merged together to filter the initial prediction set, and the genes with more than one exon were selected. This process of running AUGUSTUS with the transcriptome-derived gene set, filtering the resulting predictions and obtaining improved training parameters was repeated, till the prediction accuracy had become comparable to the average accuracy for AUGUSTUS, and the final training parameters were used with MAKER2 pipeline (Figure 9).

### 4.6. MAKER Annotation

MAKER2 release 2.31.8 was used for producing the final annotation of the larch genome (parameters for MAKER configuration are presented in Text S1, Supplementary Materials). For BLAST, the ncbi-blast-2.2.29+ version was used. The Siberian larch transcriptome data and public transcript assemblies from the related conifer species (*Gnetum gnemon, Picea abies*, *Pinus lambertiana*, *Podocarpus macrophyllus*, *Pseudotsuga menziesii*, and *Pinus taeda*), deposited in the PlantGenIE project website (https://plantgenie.org, accessed on 1 August 2018), were used as supporting evidence. Uniprot was used as the protein reference database.

The genome annotation, using MAKER2, was performed on a supercomputer segment with 56 IBM BladeCenter HS21 servers (16 GB of RAM per server) at the Department of High-Performance Computing, Institute of Space and Information Technologies, Siberian Federal University, Krasnoyarsk, Russia and took 22 days (excluding the AUGUSTUS setup and the redo database setup). The whole process involved 448 cores at 2.3 GHz/core and 896 GB of RAM with the average processor load of about 61%.

### 4.7. Assembly Evaluation and Functional Annotation

The assessment of gene completeness was completed using the BUSCO v4.0.5 benchmarking tool [49] with embryophyta reference database and protein sequences derived from the MAKER2 annotation for Siberian larch genome. The protein sets for the other gymnosperm species were taken from the treegenes database (https://treegenesdb.org, accessed on 20 June 2021).

It was shown that the distant homologs are usually more likely to be identified when using a smaller database [151] Thus, the NCBI GenBank *nr* database used to map the gene models and infer a high confidence gene set was filtered by taxonomy ID at embryophyta level. The search for the protein domains was completed using InterProScan on EMBL-EBI webserver. The GO mapping was performed using Blast2GO software integrated within OmixBox Base Platform. All of the predicted genes were mapped to NCBI GenBank *nr* database using blastp, and matches to bacteria, fungi, and archea were discarded (evalue < 1 × 10^−5^, pident > 20, qcovhsp > 20) to eliminate the genes that could potentially represent proteins other than plant genome-derived.

To compare the larch with other gymnosperm and angiosperm species, genome annotations of five other conifers, *Picea glauca*, *Picea abies*, *Pinus lambertiana*, *Pinus taeda*, and *Pseudotsuga menziesii*, and five angiosperms, *Betula pendula*, *Fagus sylvatica*, *Populus trichocarpa*, *Quercus robur*, and *Vitis vinifera*, were used to perform the blast2go GO mapping. To identify the GO terms in which the number of mapped genes differed significantly, a test of proportions was used. Two methods, based on the false discovery rate (FDR) estimation, were used for multiple comparison to correct *p*-values, according to Benjamini and Hochberg [152] and Storey [153], respectively (Figure S8, Supplementary Materials).

### 4.8. LTR-RT Insertion Time Estimation

There are two common methods for estimating the insertion time of LTR-RTs by (1) measuring the sequence divergence between two flanking LTRs and inferring their divergence time using the species-specific mutation rate, and (2) analyzing the pairwise genetic distances between the RT-encoding sequences that belong to the paralogous elements of the same monophyletic RT group. Whilst they may give different time estimates for some of the LTR lineages, the time distribution profiles produced by both methods are similar [71].

For additional de novo identification of the LTR-RT elements, LTRharvest [43] was used with options “-tis -suf -lcp -des -ssp -sds -dna”. To filter the potential false-positive hits and retrieve a stricter set of LTR-RTs, LTR_retriever [44] was used on the results of LTRharvest with the following settings “-u 1.57e-8 -missrate 0.4 -noanno”.

Zhou et al. [81] have carried out a thorough search for LTR-RTs and generated an LTRs resource for 301 plant genomes, including publicly available draft genomes of 10 gymnosperms. For the *L. sibirica* draft genome, they identified 367 LTR-RT elements. This LTR-RT library was checked against the resulted LTR-RTs found by LTRharvest and LTR_retriever using blastn, and the sequences without matches were added to the final LTR-RT database for *L. sibirica*. The final combined library for *Larix* and LTR-RT libraries for other gymnosperm species from Zhou et al. (2021) were used for the insertion time estimation. The sequence divergence was calculated, using the Jukes–Cantor model [44]:

(1) $$T=\frac{d}{2\mu },d=-\frac{3}{4}\ln\left(1-p\frac{4}{3}\right),$$

where *d* is the Jukes–Cantor genetic distance (proxy of divergence rate); *μ*—the mutation rate; and *p*—the proportion of sequence differences (*p =* 1—*identity*, where *identity* is approximated using blastn). Insertion times converted into million years assuming a synonymous substitution rate *μ* = 1.57 × 10^−8^ per site per year [154].

## 5. Conclusions

Despite being fragmented and incomplete, draft assemblies and annotations of conifer genomes still represent a valuable resource for further genomic and genetic studies. The current state of the genome annotations allows the differences between the gymnosperm and angiosperm species to be compared on a genomic level, evidenced by differences in gene abundance in different functional categories, such as cell wall organization and metabolism, PCD, and autophagy, which are related to frost resistance, seasonal senescence, stress hormone biosynthesis, and regulatory pathways.

## Figures and Tables

**Table 1:** Summary of genome assembly and gene annotation statistics for Siberian larch genome.

Parameter	*Larix sibirica*
Number of chromosomes	12
Estimated genome size (1C), Gbp	12.03 (12.30 pg) ^1^
Assembly length, Gbp	5.59 ^2^/12.34 ^3^
Assembly N50, bp	3098 ^2^/6443 ^3^
GC content, %	35.41
Repeat content, %	65.98
Number of predicted gene models	39,370
Number of full-length gene models	24,551
Average CDS length, bp	244.29
Average intron length, bp	360.93
Longest intron length, bp	10,153

^1^ https://cvalues.science.kew.org/search/gymnosperm (accessed on 25 July 2022); ^2^ based on contigs without gaps; ^3^ based on scaffolds with gaps.

**Table 2:** The summary statistics of the Siberian larch assembly with a minimum contig length of 200 bp.

Assembly	Number, mln	N50, bp	Maximum Length, bp	Total Length, Gbp
Contigs	12.40	1074	128,642	7.99
Scaffolds	11.33	6443	354,326	12.34

## Data Availability

Data generated during the study, including gff annotation files, RepeatModeler generated and combined plant repeat libraries, RepeatMasker output, LTR-RT non-redundant library, and LRR-containing sequences, are available in figshare with DOI 10.6084/m9.figshare.19785913 or in SibFU repository at https://hpccloud.sfu-kras.ru/owncloud/index.php/s/GMBabOGEgqOD4JX (accessed on 12 July 2022). Transcriptome sequencing data from bud, needle, stem, seedling, and cambium tissues are available at NCBI sequence read archive under accession numbers SRX9464971, SRX14986114, SRX14997110, SRX14997111, and SRX14997112. Transcriptome assemblies are available under accession numbers GIXH00000000, GJYD00000000, GJYL00000000, GJYN00000000, and GJYW00000000.

## Supplementary Materials

The following supporting information can be downloaded at: https://www.mdpi.com/article/10.3390/plants11152062/s1, Text S1: Parameters for MAKER configuration; Table S1: Summary of repeat content in Siberian larch genome assembly annotated using RepeatMasker and combined library, comprising the RepeatModeler-derived library classified with TEclass, RepBase, MIPS, CPRD and PIER v1.0 libraries; Table S2: Summary of repeat content in a portion of long nanopore reads annotated using RepeatMasker and combined library, comprising the RepeatModeler-derived library classified with TEclass, RepBase, MIPS, CPRD and PIER v1.0 libraries; Table S3: LTR repeat superfamilies in the loblolly pine BAC library found in the Siberian larch genome; Table S4: The number of transcripts containing the LRR domain among all transcripts, and the number of transcripts containing the ARC domain among transcripts shorter than 850 bp for transcriptomes of different Siberian larch tissues; Table S5: Assessment of gene space completeness using BUSCO; Table S6: Assessment of gene space completeness using BUSCO protein mode and protein sequences for annotated gene models; Table S7: Summary of gene and genome assembly statistics among conifer and angiosperm plant species; Table S8: GO terms associated with cell wall metabolism and organization, programmed cell death (PCD), and hormone response that had a higher number of annotated genes in gymnosperms than in angiosperms; Figure S1: Length distribution of the main LTR-retrotransposons families in the Siberian larch genome; Figure S2: Length distribution of the Gypsy-retrotransposon superfamilies in the Siberian larch genome; Figure S3: Length distribution of the Copia-retrotransposon superfamilies in the Siberian larch genome; Figure S4: Length distribution of the main nonLTR-retrotransposon families in the Siberian larch genome; Figure S5: Length distribution of the main DNA-transposon families in the Siberian larch genome; Figure S6: (**A**–**E**)—transcripts with LRRs found using nine LRR families (lrr1-lrr9) in the transcriptomes of five tissues (for example, 43 transcripts were found by each of the LRR-1, LRR-4 and LRR-8 families), (**F**)—the distribution of the lengths of the amino acids sequences of the putative R-genes, LRR—transcripts containing the LRR domain (in blue), LRR and ARC—transcripts containing the LRR and ARC domains (in red); Figure S7: Repeats overlapping with predicted gene models. (**A**)—number of repeats overlapping with coding parts (CDS) with at least 20% overlap, (**B**)—the most frequent functional annotations of genes that overlap with repeats; Figure S8: Storey’s q-value estimates for FDR in GO comparation. (**A**)—the estimated proportion of true null hypotheses (π_0_) vs. λ; (**B**)—the number of significant tests vs. each q-value cutoff; (**C**)—the q-values vs. the *p*-values; (**D**)—the number of expected false positives vs. the number of significant tests.
