# Supplementary material for: Annotation of Siberian Larch (Larix sibirica Ledeb.) Nuclear Genome—One of the Most Cold-Resistant Tree Species in the Only Deciduous GENUS in Pinaceae
Source: Plants (Basel). 2022 Aug 6;11(15):2062. doi: 10.3390/plants11152062 (PMC9370799; doi:10.3390/plants11152062)
Supplement: Supplementary file 1 [file plants-11-02062-s001.zip › plants-1809628-supplementary.pdf]

Text S1. Parameters for MAKER configuration.

genome=~/conifer/larch-c-v2.fa

organism_type=eukaryotic

est=~/test_makerMPI/Trinity.fasta #non-redundant set of assembled ESTs in fasta format (classic EST analysis)

altest=UCPtaeda.fa,UCGgnemon.fa,UCPabies.fa,UCPlambertiana.fa,UCPmacrophyllus.fa,UCPmenziesii.fa #EST/cDNA sequence file in fasta format from an alternate organism

est_gff=~/conifer/larch-cufflinks.gtf #EST evidence from an external gff3 file

protein=~/uniprot_sprot.fasta #protein sequence file in fasta format

model_org=all #select a model organism for RepBase masking in RepeatMasker

rmlib=larch-rep2.fa #provide an organism specific repeat library in fasta format for RepeatMasker

softmask=1 #use soft-masking rather than hard-masking in BLAST (i.e. seg and dust filtering)

augustus_species=larch4 #Augustus gene prediction species model

Table S1. Summary of repeat content in Siberian larch genome assembly annotated using RepeatMasker and combined library, comprising the RepeatModeler-derived library classified with TEclass, RepBase, MIPS, CPRD and PIER v1.0 libraries.

Superfamily	Length, bp	Number	% of genome length
**Class I: LTR retrotransposon**			
Gypsy	192527487	878785	1.60
Gypsy/PtTalladega	1663158	3646	0.01
Gypsy/IFG	254098	990	<0.01
Gypsy/PtAppalachian	208844	773	
Gypsy/PtOuachita	191626	1025	
Gypsy/Gymny	138565	476	
Gypsy/PtBastrop	94602	594	
Gypsy/RLG	62107	1004	
Gypsy/PtOzark	24665	234	
Gypsy/ALISEI	21741	209	
Gypsy/PsAppalachian	16203	108	
Gypsy/PtAngelina	497	4	
**Total Gypsy**	**195203593**	**887848**	**1.63**
Copia	125043599	627489	1.04
Copia/PtConagree	115681	731	**<0.01**
Copia/Silava_Pta	115133	581	
Copia/PtPineywoods	64007	401	
Copia/PtCumberland	32521	251	
**Total Copia**	**125370941**	**629453**	**1.04**
DIR	7936310	51295	0.07
ERV	444525	7717	<0.01
BEL	418006	7389	
DIRS	135235	2287	
ATGP	22493	272	
RNLTR	10324	197	
RMER	6732	110	
RIRE	6682	100	
Unclassified LTR	1703439143	6246018	14.19
**Total LTR**	**2032993984**	**7832686**	**16.94**
**Class I: Non-LTR retrotransposon**			
LINE	1124723825	4237879	9.37
LINE/I	265525048	1577725	2.21
LINE/L1	21091550	94925	0.18
**Total LINE**	**1411340423**	**5910529**	**11.76**
Penelope	15970080	79330	0.13
Penelope/Poseidon	39219	411	<0.01
**Total Penelope**	**16009299**	**79741**	**0.13**
SINE	10566091	79177	0.09
LOA	19191	275	<0.01
Jockey	15690	120	
TRAS	14525	228	
LIN#_SM	10491	169	
BovB	9128	155	

Superfamily	Length, bp	Number	% of genome length
Outcast	6887	121	
Hero	6599	122	
Other non-LTR	22649056	133018	0.19
**Total non-LTR**	**1460647380**	**6203655**	**12.17**
Other retrotransposons	390376007	1771180	3.25
**Total Class I**	**3884017371**	**15807521**	**32.36**
**Class II: DNA transposon**			
TIR	19582568	105413	0.16
Helitron	6739134	28439	0.06
EnSpm	2142128	28885	0.02
MuDR	587040	8191	<0.01
MuDR/Vandal	29783	546	
MuDR/Rehavkus	15474	220	
MuDR/Arnold	13638	151	
MuDR/ATMU	8652	143	
MuDR/OSMU	6609	85	
**Total MuDR**	**661196**	**9336**	**<0.01**
Mariner	462692	7270	<0.01
REP	167449	1355	
Maverick	517499	5211	
hAT	1073811	15821	
Mite	32189	505	
TcMar	30674	446	
Harbinger	20160	316	
Stowaway	3957	72	
PiggyBac	1065	20	
Tourist	845	16	
Unclassified	539184594	2226370	4.49
**Total Class II**	**570619961**	**2429475**	**4.76**
**Others**			
Unclassified	262494312	1345055	2.19
Simple repeats	46499317	983566	0.39
Host	12847092	18499	0.11
Low complexity	11560642	211886	0.09
Other	1215941	76131	0.01
tRNA	299376	4982	<0.01
rRNA	189617	1947	
RNA	1325	16	
Caulimovirus	401990	3767	<0.01
**Grand Total**	**4790146944**	**20882845**	**39.91**

Table S2. Summary of repeat content in a portion of long nanopore reads annotated using RepeatMasker and combined library, comprising the RepeatModeler-derived library classified with TEclass, RepBase, MIPS, CPRD and PIER v1.0 libraries.

Class	Number	Length, bp	% of long read length
LTR	6062560	2996181397	41.39
NonLTR	3851106	1236818752	17.08
Unclassified Retro	2144145	640871730	8.85
DNA	2220166	912053154	12.60
Unclassified Mobile DNA	718063	244714706	3.38
Simple	567070	29625299	0.41
Low_complexity	90295	5201845	0.07
Host	10475	2401744	0.03
RNA	3801	597481	0.01
Caulimovirus	2615	227664	0.00
**Total**	**15670296**	**6068693772**	**83.83**

Table S3. LTR repeat superfamilies in the loblolly pine BAC library found in the Siberian larch genome.

Superfamily		Number of copies	Length, bp
Gypsy	PtTalladega	3646	1663158
	IFG	990	254098
	PtAppalachian	773	208844
	PtOuachita	1025	191626
	Gymny	476	138565
	PtBastrop	594	94602
	PtOzark	234	24665
	PsAppalachian	108	16203
	PtAngelina	4	497
Copia	PtConagree	731	115681
	Silava_Pta	581	115133
	PtPineywoods	401	64007
	PtCumberland	251	32521

Table S4. The number of transcripts containing the LRR domain among all transcripts, and the number of transcripts containing the ARC domain among transcripts shorter than 850 bp for transcriptomes of different Siberian larch tissues.

**Sample**	**Total number of transcripts**	**Number of sequences containing LRR**	**Number of sequences containing LRR, %**	**Number of transcripts > 850 bp**	**Number of transcripts containing ARC domain**
Shoot	129,220	1,846	1.43	182	56
Seedling	55,584	733	1.32	14	5
Cambium	122,115	1,599	1.31	82	18
Autumn bud	22,116	194	0.88	4	2
Needles	20,276	110	0.54	-	-

Table S5. Assessment of gene space completeness using BUSCO.

**Species**	**Complete, %**	**Partial, %**	**Complete and partial, %**	**Mapped to genome, %**
Pinus taeda genes*	7.0	2.2	9.2	-
Picea abies genes	22.5	13.1	35.6	-
Picea glauca genes	17.2	9.1	26.3	-
Larix sibirica genes	17.5	12.3	29.8	-
Picea abies proteins**	28.1	27.3	55.4	-
Picea glauca proteins	21.1	11.5	32.6	-
Pinus taeda proteins	41.7	19.4	61.1	-
Pinus lambertiana proteins	73.4	7.5	80.9	-
Pseudotsuga menziesii proteins	68.5	11.8	80.3	-
Abies alba proteins	15.8	17.9	33.7	-
Larix sibirica proteins	19.6	19.0	38.6	-
Picea abies genome	30.1	16.0	46.1	-
Picea glauca genome	27.4	17.5	44.9	-
Pinus taeda genome	28.6	14.5	43.1	-
Larix sibirica genome***	27.5	17.8	45.3	-
Larix sibirica mRNA	18.8	17.0	35.8	-
Larix sibirica transcriptome (bud)	24.4	19.9	44.3	88.8
Larix sibirica transcriptome (needle)	12.3	12.4	24.7	85.0
Larix sibirica transcriptome (cambium)	62.4	15.6	78.0	77.9
Larix sibirica transcriptome (shoot)	84.5	6.1	90.6	88.7
Larix sibirica transcriptome (seedling)	45.0	20.6	65.6	81.9

* Using BUSCO genome mode and nucleotide sequences for annotated gene models
** Using BUSCO protein mode and protein sequences for annotated gene models
*** Using BUSCO genome mode and whole genome assemblies

Table S6. Assessment of gene space completeness using BUSCO protein mode and protein sequences for annotated gene models.

Species	Complete, %	Partial, %	Complete and partial, %
Pinus lambertiana	73.4	7.5	80.9
Pseudotsuga menziesii	68.5	11.8	80.3
Pinus taeda	41.7	19.4	61.1
Picea abies	28.1	27.3	55.4
Larix sibirica	19.6	19.0	38.6
Abies alba	15.8	17.9	33.7
Picea glauca	21.1	11.5	32.6

Table S7. Summary of gene and genome assembly statistics among conifer and angiosperm plant species

**Parameter**	***Larix sibirica*** (this study)	***Pinus taeda*** (Wegrzyn et al., 2014)	***Picea abies*** (Nystedt et al., 2013)	***Picea glauca*** (Warren et al., 2015)	***Populus trichocarpa*** (Tuskan et al. 2006)*
Estimated genome size, Gbp (Pellicer & Leitch, 2020)	12.03	20.15	19.57	15.79	0.48
Assembly length**, Gbp	5.59 / 12.34	20.43 / 22.10	9.99 / 12.30	20.78 / 25.47	0.42
Assembly N50**, Kbp	3,098 / 6,443	100,218 / 110,557	7,747 / 5,206	34,405 / 46,559	-
Number of chromosomes	12	12	12	12	19
GC content (%)	35.41	38.06	38.81	37.08	
Repeat content (%)	65.98	81.8	70.0***	-	41
Number of predicted gene models	39,370	50,172	70,968	102,915	41,377
Number of full-length gene models	24,551	-	-	-	-
Average CDS length, bp	244.29	419.81	287.21	283.56	233.05
Average intron length, bp	360.93	1146.12	997.94	642.73	468.08
Maximum intron length, bp	10,153	568 968	68 268	44 113	96 842

* assembly at chromosome level

** without gaps / with gaps.

*** inferred from unassembled reads (Nystedt et al., 2013)

Table S8. GO terms associated with cell wall metabolism and organization, programmed cell death (PCD), and hormone response that had a higher number of annotated genes in gymnosperms than in angiosperms.

Category	GO.IDs	GO.Names
Cell wall and organization	GO:0009094	L-phenylalanine biosynthetic process
	GO:0009505	plant-type cell wall
	GO:0009664	plant-type cell wall organization
	GO:0009698	phenylpropanoid metabolic process
	GO:0009699	phenylpropanoid biosynthetic process
	GO:0016998	cell wall macromolecule catabolic process
	GO:0030245	cellulose catabolic process
	GO:0042546	cell wall biogenesis
	GO:0046274	lignin catabolic process
	GO:0047782	coniferin beta-glucosidase activity
	GO:0050269	coniferyl-aldehyde dehydrogenase activity
	GO:0071555	cell wall organization
PCD	GO:0006914	autophagy
	GO:0010506	regulation of autophagy
	GO:0012501	programmed cell death
	GO:0012502	induction of programmed cell death
Hormone response	GO:0009695	jasmonic acid biosynthetic process
	GO:0009723	response to ethylene
	GO:0009725	response to hormone
	GO:0009738	abscisic acid-activated signaling pathway
	GO:0009753	response to jasmonic acid
	GO:0010427	abscisic acid binding
	GO:0038199	ethylene receptor activity
	GO:0051740	ethylene binding

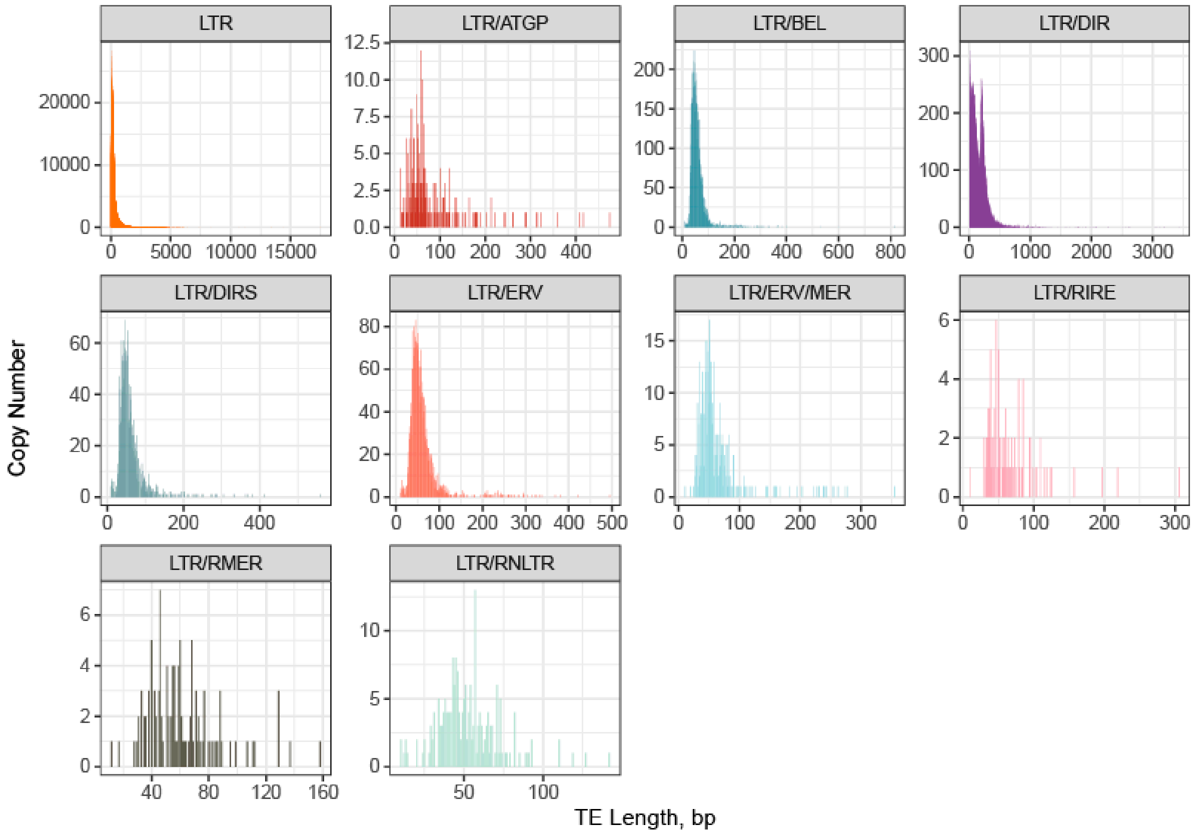

Figure S1. Length distribution of the main LTR-retrotransposons families in the Siberian larch genome.

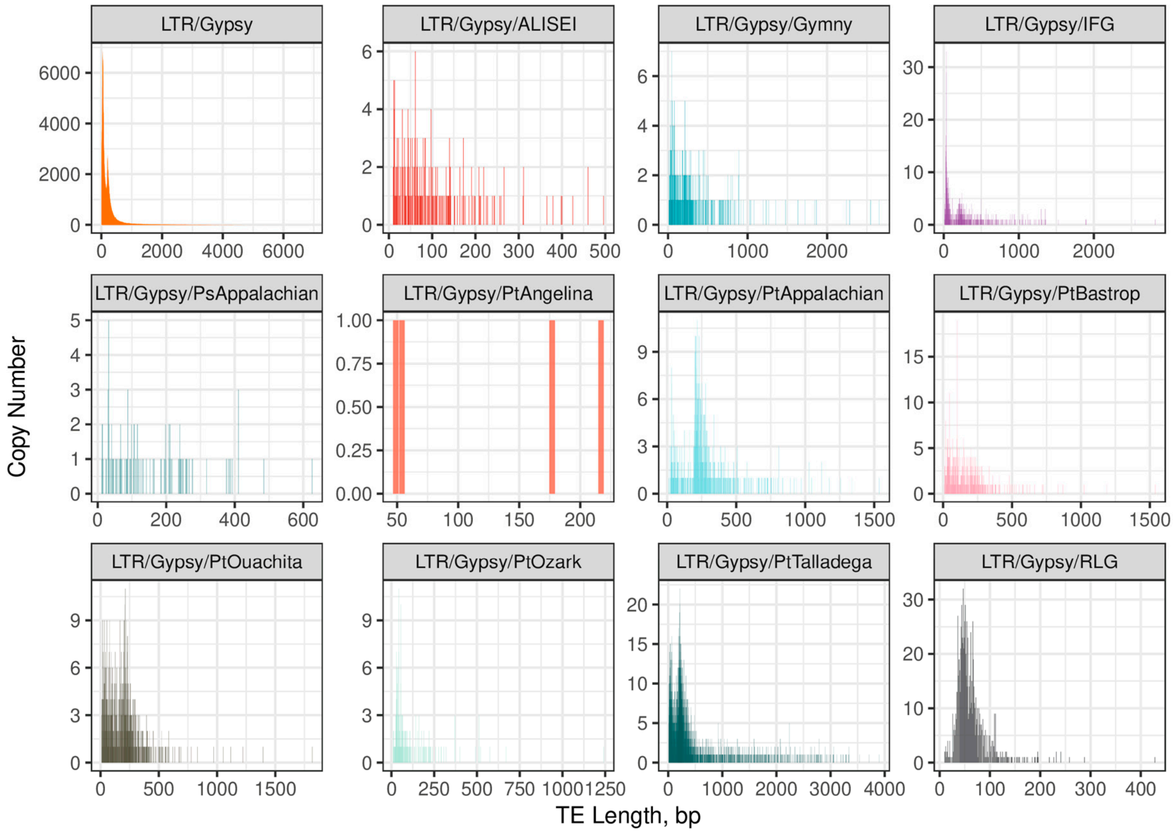

Figure S2. Length distribution of the Gypsy-retrotransposon superfamilies in the Siberian larch genome.

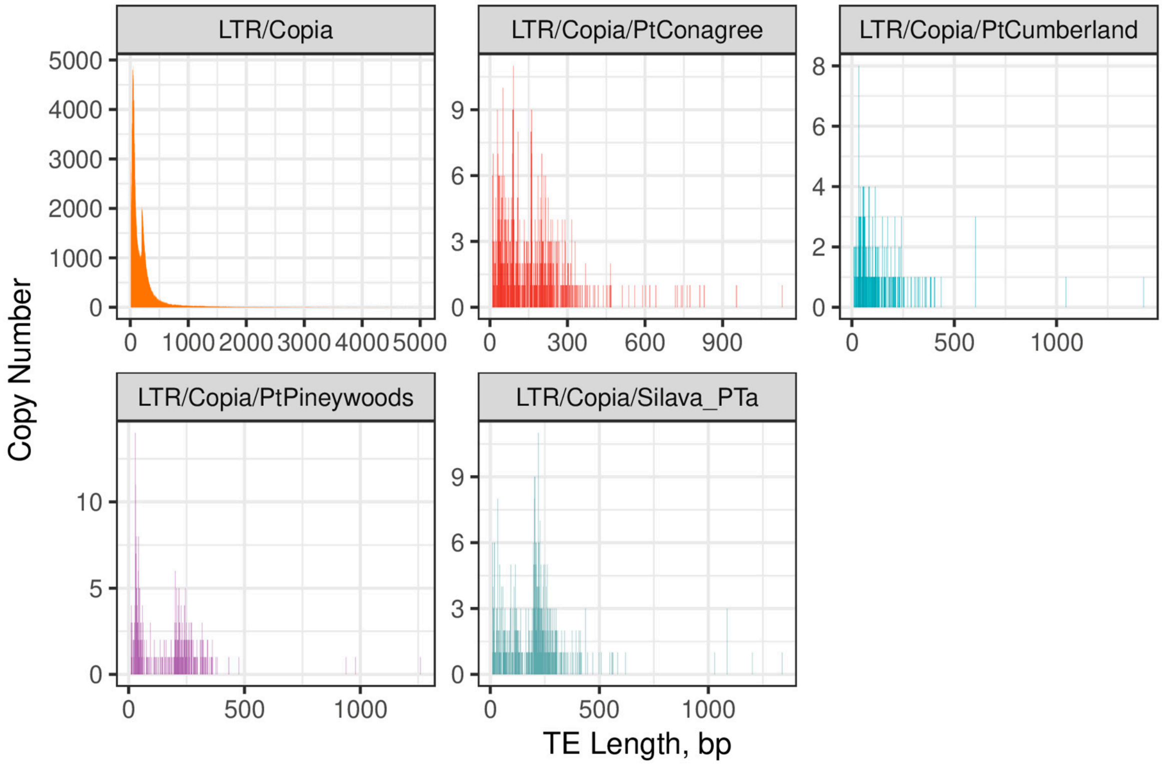

Figure S3. Length distribution of the Copia-retrotransposon superfamilies in the Siberian larch genome.

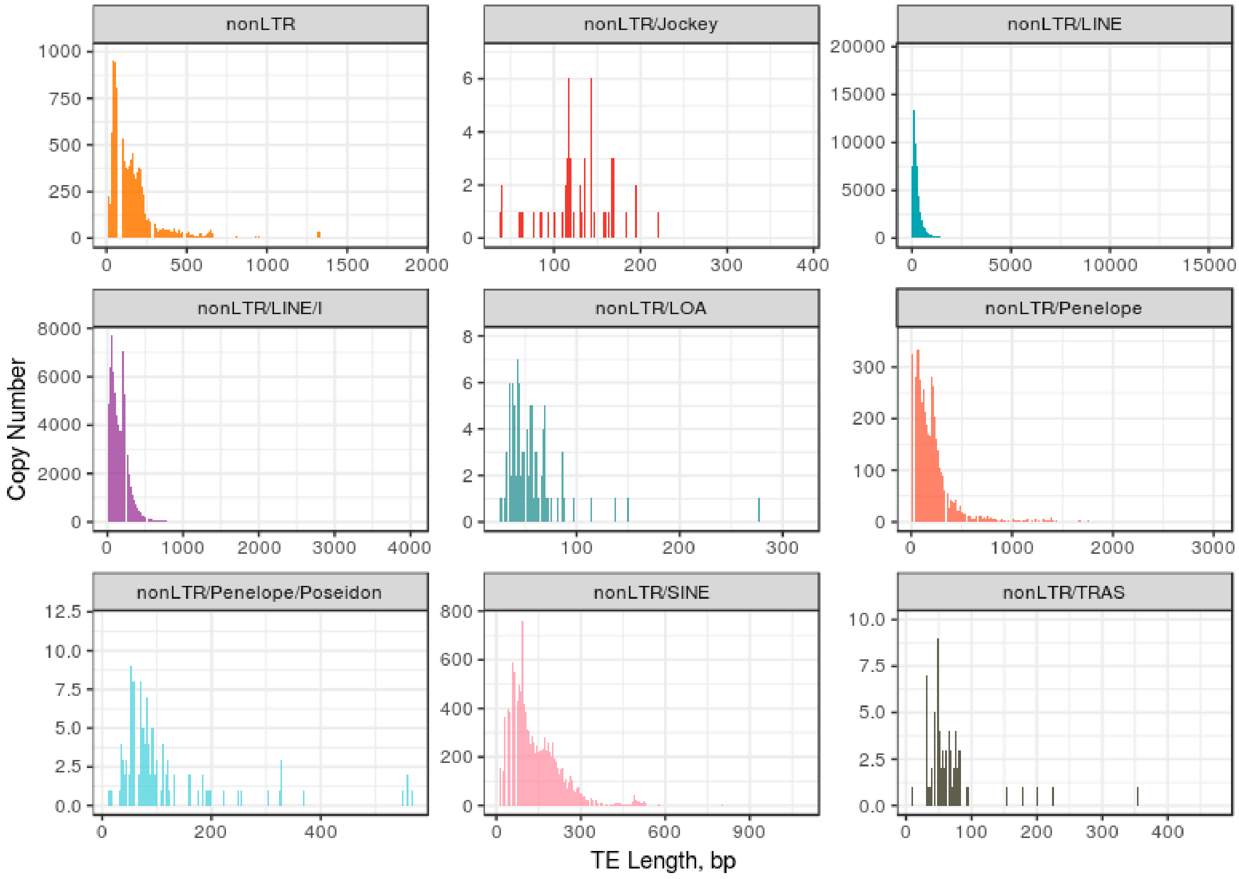

Figure S4. Length distribution of the main nonLTR-retrotransposon families in the Siberian larch genome.

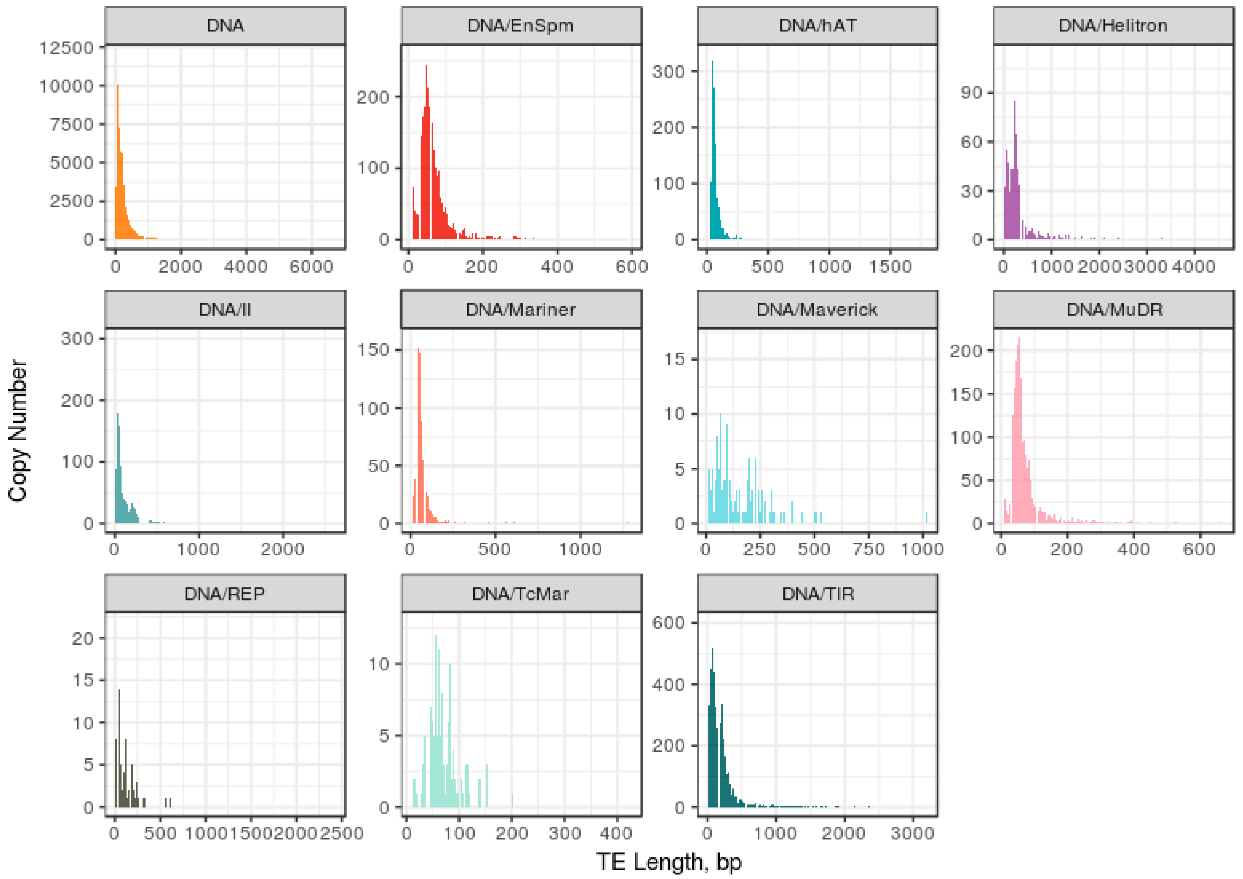

Figure S5. Length distribution of the main DNA-transposon families in the Siberian larch genome.

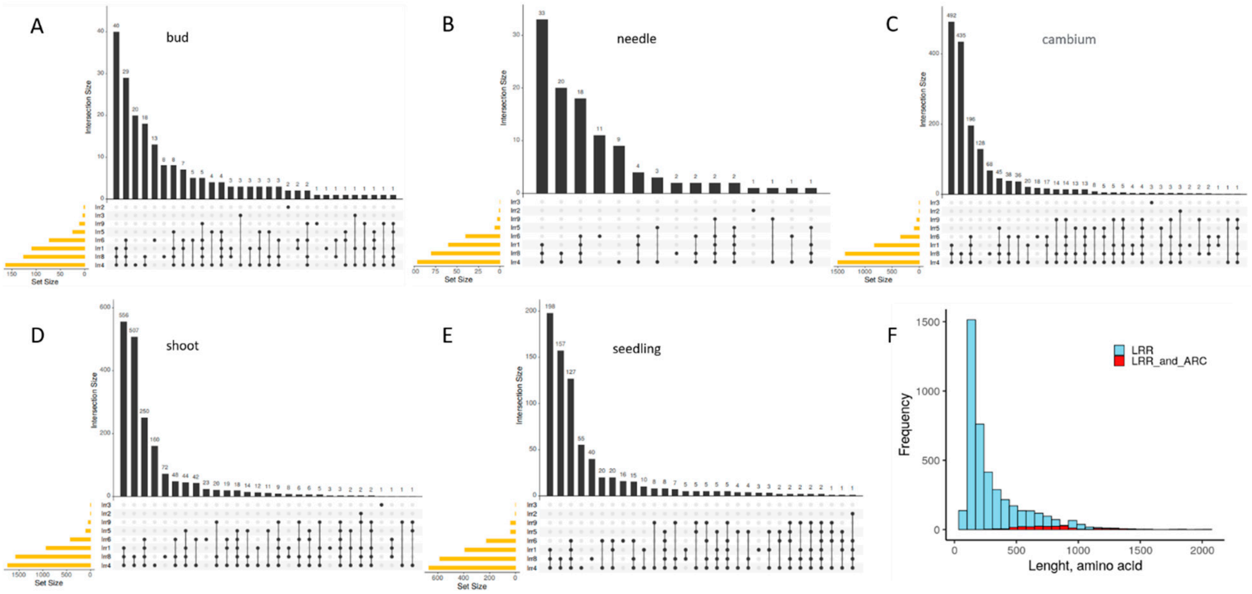

Figure S6. A-E - transcripts with LRRs found using nine LRR families (lrr1-lrr9) in the transcriptomes of five tissues (for example, 43 transcripts were found by each of the LRR-1, LRR-4 and LRR-8 families), **F -** the distribution of the lengths of the amino acids sequences of the putative R-genes, LRR — transcripts containing the LRR domain (in blue), LRR and ARC — transcripts containing the LRR and ARC domains (in red).

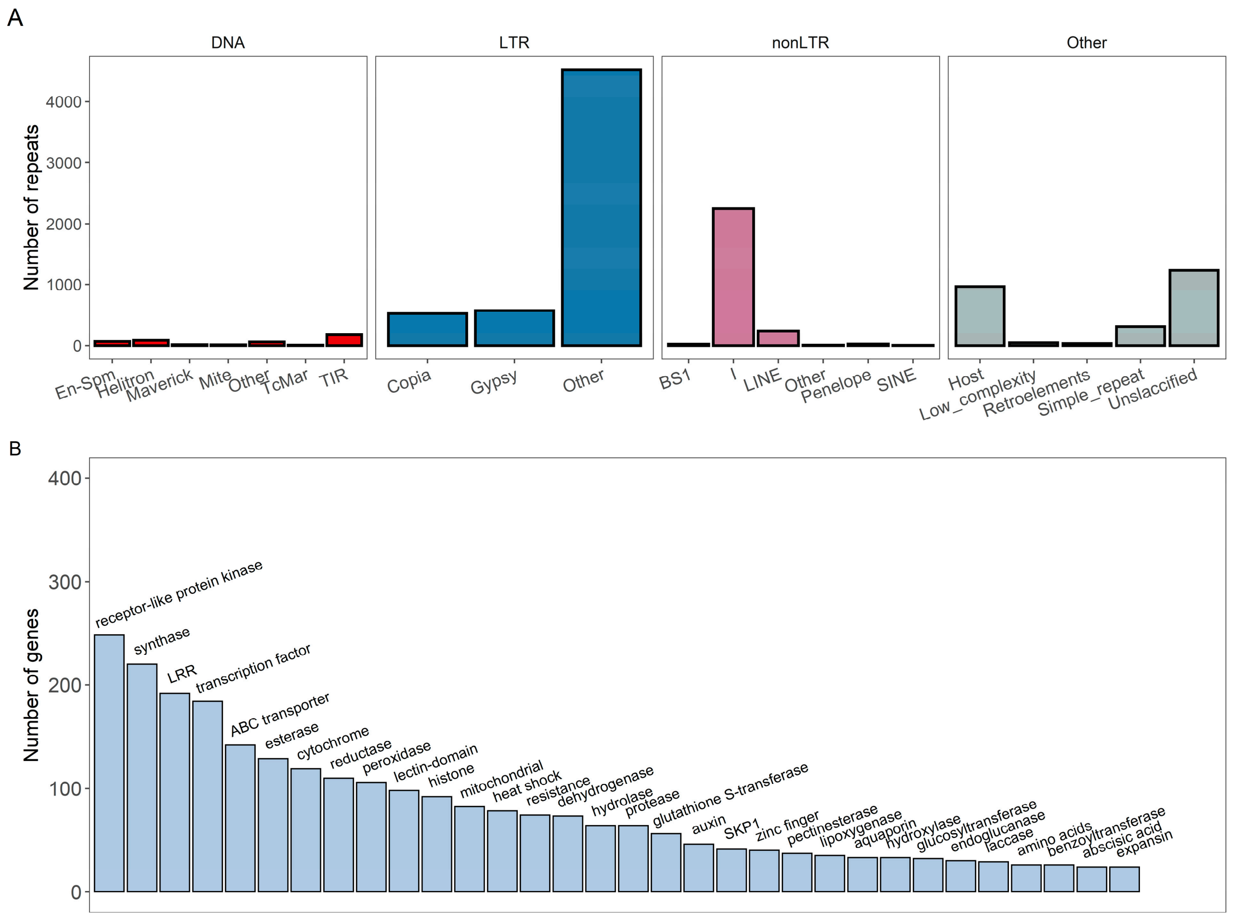

Figure S7. Repeats overlapping with predicted gene models. **A -** number of repeats overlapping with coding parts (CDS) with at least 20% overlap, **B** – the most frequent functional annotations of genes that overlap with repeats.

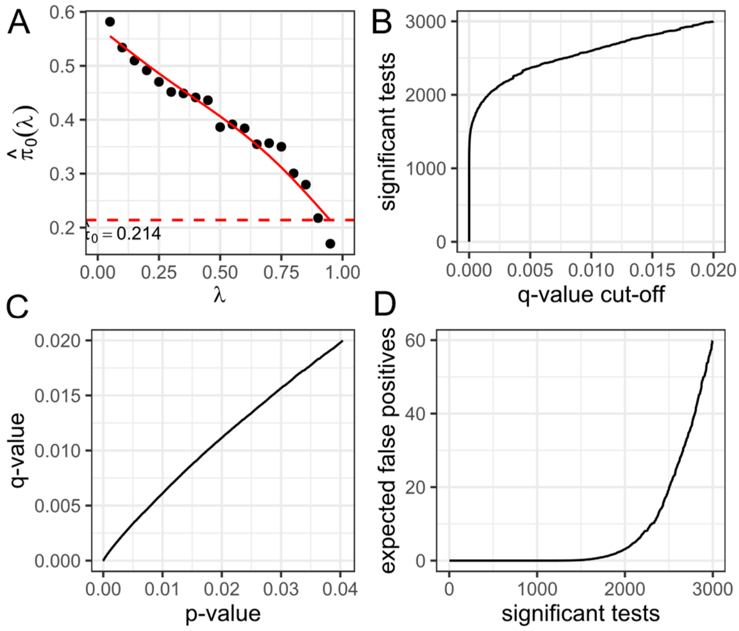

Figure S8. Storey's q-value estimates for FDR in GO comparation. **A -** the estimated proportion of true null hypotheses (π_0) vs λ, **B -** the number of significant tests vs each q-value cutoff, **C -** the q-values vs the p-values, **D -** the number of expected false positives vs the number of significant tests.
